# β-Lactamase-activated antimicrobial dendron *via* the amine uncaging strategy†

**DOI:** 10.1039/d5sc02412a

**Published:** 2025-05-12

**Authors:** Hao Luo, Zeyu Shao, Karen Hakobyan, Jiangtao Xu, Rhiannon P. Kuchel, Shyam Kumar Mishra, Mark Willcox, Edgar H. H. Wong

**Affiliations:** a School of Chemical Engineering, University of New South Wales Sydney NSW 2052 Australia; b Electron Microscope Unit, University of New South Wales Sydney NSW 2052 Australia; c School of Optometry and Vision Science, University of New South Wales Sydney NSW 2052 Australia edgar.wong@unsw.edu.au

## Abstract

The development of new antimicrobial agents to combat multidrug-resistant (MDR) bacteria, especially those that produce β-lactamase enzymes, is a critical step in preventing a post-antibiotic era. Herein, we develop a new membrane-active antimicrobial prodrug (BLM-Dendron) based on the amine uncaging strategy (AUS) whereby the amine groups of a cationic amphipathic dendron are caged/masked initially but can be uncaged specifically in the presence of β-lactamase enzymes (*e.g.*, penicillinase) to enable precise antimicrobial activation. BLM-Dendron undergoes self-assembly in water to form spherical nanoparticles with average hydrodynamic diameter (*D*_H-avg_) of *ca.* 200 nm and is bacteriostatic against (MDR) *P. aeruginosa*, *E. coli* and *S. aureus* in the presence of penicillinase. In addition, the uncaged dendron also has bactericidal and antibiofilm activities against wild-type *P. aeruginosa*. For instance, once uncaged, the dendron has the capacity to eliminate ≥99.99999% of planktonic cells after 24 h of treatment. Mechanistic studies show that the activated dendron is indeed membrane-active and disrupts the inner and outer membranes of bacteria cells. Notably, the prodrug BLM-Dendron has excellent hemocompatibility (at least 3.6 times higher) and low cytotoxicity (at least twice better) compared to the original molecule with exposed cationic groups. This study importantly demonstrates the benefit of using AUS to bestow cationic amphipathic antimicrobial agents with higher biocompatibility and targeted activation capabilities, as these features are key for translation into clinical settings.

## Abstract 2

Overcoming the toxicity and selectivity issues of cationic antimicrobial peptide mimics *via* amine-caged prodrugs that could be specifically uncaged by bacteria-derived β-lactamase enzyme.

## Graphical abstract

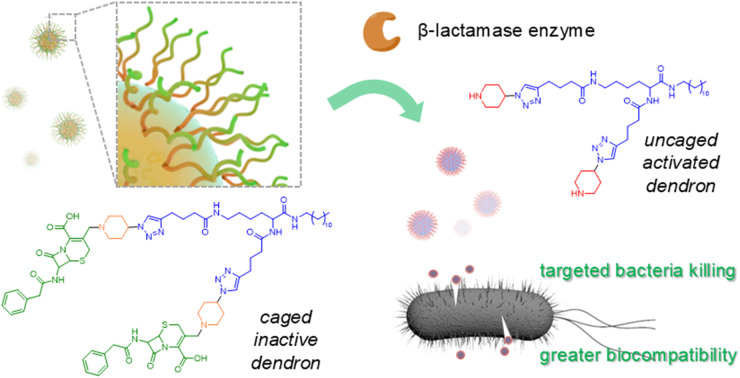

## Introduction

Antimicrobial resistance (AMR) is a global threat to public health given the rise of infections caused by multidrug-resistant (MDR) bacteria in recent years.^1,2^ The World Health Organization (WHO) has warned that deaths caused by AMR-related diseases could amount to 10 million people per annum by 2050 if no new solutions are found.^3^ Apart from threatening healthcare and the global economy, AMR also negatively impacts agriculture and food security due to significant rates of animal mortality.^4^ For some time, the pathogens that have been classified in the ‘Critical Group’ by WHO as a top priority to overcome are all Gram-negative bacteria that are resistant to the β-lactam antibiotic family, and the next ‘High Group’ priority list contains two more such pathogens (*i.e.*, carbapenem-resistant *Pseudomonas aeruginosa* and cephalosporin-resistant *Neisseria gonorrhoeae*).^3^ These bacteria are resistant because they produce β-lactamase enzymes that render the antibiotics ineffective *via* the opening of the β-lactam ring through hydrolysis.^5^ Therefore, it comes as no surprise that the most intuitive strategy to combat these bacteria to date is to employ a cocktail of β-lactam antibiotics and β-lactamase inhibitors, in which the latter serve to protect and preserve the activity of the former by blocking and/or deactivating the enzymes.^6^ Currently, there are around nine known β-lactamase inhibitors approved for use in combination with β-lactam antibiotics (*e.g.*, Xacduro and Zerbaxa).^6,7^ However, resistant strains are beginning to emerge to counteract the antibiotic-enzyme inhibitor combinations by increasing enzyme levels and efflux pumps.^8–11^

A promising class of compounds that hinders resistance development in bacteria are antimicrobial peptides (AMPs), and mimics thereof. These compounds could prove advantageous in combating MDR bacteria due to their ability to impart activity *via* a multimodal mechanism, including the widely known membrane disruption pathway.^12–24^ From a general molecular perspective, the key functional groups that bestow this class of compounds with their excellent antimicrobial activity are typically a combination of cationic (amine) and hydrophobic groups. Despite their potential, this class of compounds has unfortunately had limited success in clinical trials due to toxicity and stability issues, as the same chemical structural features that give rise to antimicrobial activity can also cause off-target toxicity.^25,26^ Over the years, substantial research efforts have been undertaken to increase their specificity towards bacteria and/or decrease toxicity to mammalian cells, mostly *via* augmentation of the functional groups (*e.g.*, variation of the amount and type of cationic and hydrophobic groups, and incorporation of neutral hydrophilic groups) and architecture, or through combination therapies with other antimicrobial agents such as antibiotics.^13,27–44^ In recent times, researchers have begun developing antimicrobial polymer platforms with stimuli-responsiveness wherein the antimicrobial activity could be precisely switched on (or off) by a stimulus to better modulate the biological properties.^45–47^

**Fig. 1:**
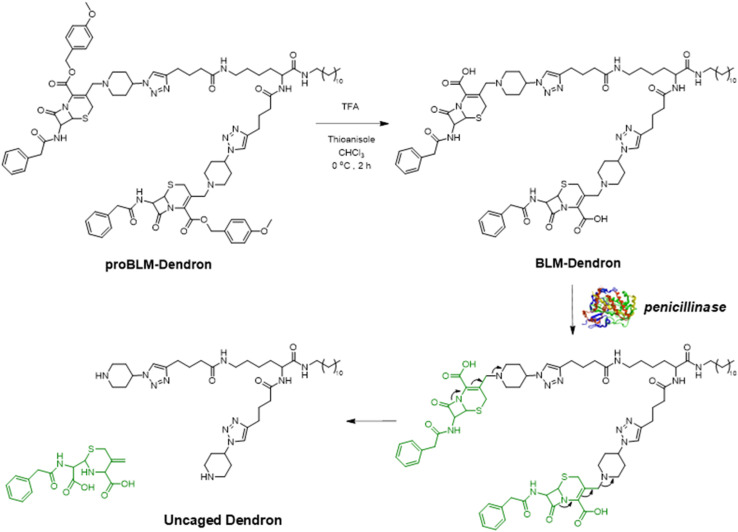
Key chemical structures of compounds used in this study. The deprotection of proBLM-Dendron leads to the amine-caged BLM-Dendron, which upon exposure to β-lactamase enzymes such as penicillinase, undergoes the postulated self-immolative elimination pathway to reveal the uncaged dendron and confer antimicrobial activity. Noteworthy, the chemical structure of Ref-Dendron is the HCl salt form of the uncaged dendron.

In the same vein, our group has been recently developing novel AMP mimics whereby the cationic amine groups are initially caged (and hence inactive and less toxic), only to be uncaged by a specific trigger (*e.g.*, light or galactosidase enzyme) to confer on-demand antimicrobial activity.^48,49^ This specific approach, coined by us as the amine uncaging strategy (AUS), focuses on addressing the root cause of toxicity since the cationic groups can bind indiscriminately with both bacteria and mammalian cells, which is often the first step preceding other molecule–cell interaction events. We have previously shown that the biocompatibility of cationic amphipathic antimicrobial macromolecules improved substantially using AUS and believe that the exploration of other triggers (uncaging chemistries) would further expand the versatility of this approach. Thus, in this study, we report a new self-assembling amine-caged dendron (BLM-Dendron) that can be uncaged in the presence of penicillinase, a β-lactamase enzyme, to reveal cationic amine groups and confer antimicrobial activity (Fig. 1). The uncaging efficiency is high, as suggested by ^1^H NMR spectroscopic analysis, and can proceed effectively in complex cell culture media to kill bacteria *in situ* (*vide infra*), which is rare given that a lot of other enzyme-responsive systems lose their activity when tested in media containing high concentrations of proteins and salts. Considering that penicillinase is only produced by pathogenic bacteria, the development of BLM-Dendron thus represents an important step towards designing biocompatible AMPs and mimics with precise and targeted bacteria killing abilities while potentially saving the human microbiome.

## Results and discussion

The AMP mimic that we chose to modify and convert into BLM-Dendron (as an example to illustrate the benefit and versatility of AUS) was a ‘Y-shaped’ antimicrobial dendron with two secondary amine units and one dodecyl tail as the cationic and hydrophobic groups, respectively. This particular AMP mimic, investigated in our recent study,^42^ has good antimicrobial activity but only moderate biocompatibility and hence is an ideal candidate to demonstrate that its biological properties can be improved upon using AUS. The AMP mimic also acted as the reference compound (duly defined herein as Ref-Dendron) when comparing the chemical and biological properties of the modified BLM-Dendron. The design of BLM-Dendron was inspired by Kelso and co-workers where β-lactam antibiotics were used to make nitric oxide prodrugs and act as trigger points to initiate the release of the gas molecules upon contact with β-lactamase enzymes,^50,51^ and also by the β-lactamase-induced disassembly of polymer self-assemblies^52^ and hydrogels.^53^ In a similar vein, the secondary amines of the AMP mimicking Ref-Dendron were caged and modified with a commercially available halide-functionalized cephalosporin antibiotic to eventually produce BLM-Dendron. It is worth mentioning that other derivatives of β-lactam precursors could also potentially be adopted.

**Fig. 2:**
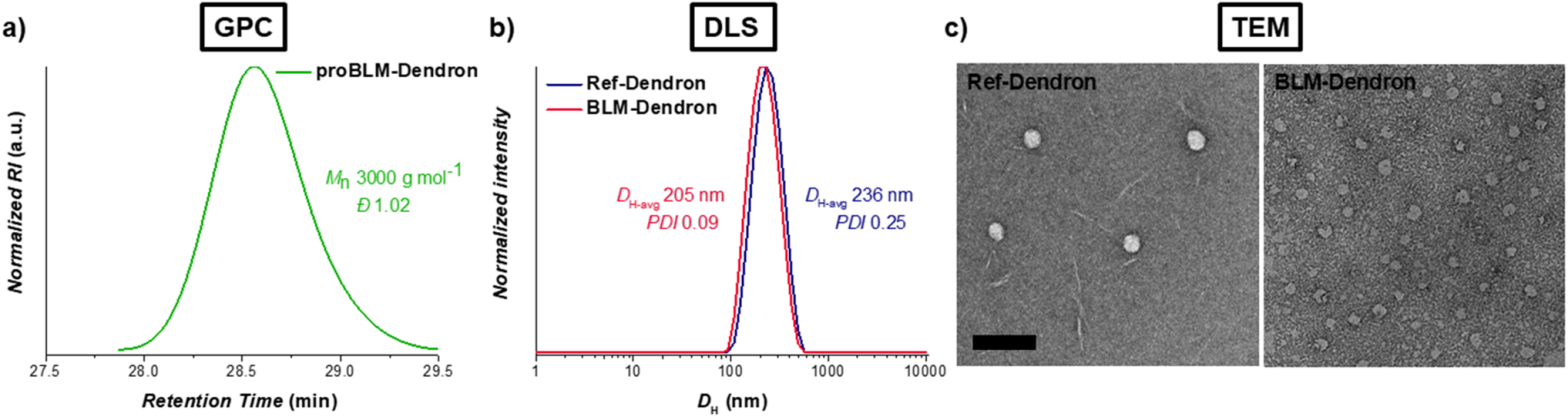
Characterization of dendrons. (a) GPC differential refractive index (RI) chromatogram of proBLM-Dendron as measured in dimethylacetamide eluent. (b) DLS traces of Ref-Dendron and BLM-Dendron in deionized water (intensity distribution *vs.* hydrodynamic diameter) at 128 μg mL^−1^. (c) TEM micrographs of Ref-Dendron (left) and BLM-Dendron (right) samples. The scale bar is 200 nm.

The synthesis leading up to the *para*-methoxybenzyl ester protected form (proBLM-Dendron) and the subsequent deprotected BLM-Dendron was straightforward, and the chemical structures of all the products and intermediates were verified by ^1^H and ^13^C NMR spectroscopic analysis (Fig. S1–S10, ESI†). For instance, the resonance due to triazolyl hydrogens was clearly visible in the NMR spectra of proBLM-Dendron and BLM-Dendron at *δ*_H_ 7.75 to 7.89 ppm, which confirmed the successful azide–alkyne cycloaddition in forming these compounds. GPC analysis was also performed on proBLM-Dendron to check its molecular weight distribution, which yielded a symmetrical distribution with a very low dispersity (*Đ*) value of 1.02, as expected of a unimolecular compound (Fig. 2a) and within deviation due to inherent column broadening effects. In addition, it is important to note that the measured number-averaged molecular weight (*M*_n_) of 3000 g mol^−1^ was relative to poly(methyl methacrylate) calibration standards and thus not absolute. The exact molecular weight of proBLM-Dendron is 1655 g mol^−1^.

Given the amphipathic nature of BLM-Dendron and Ref-Dendron, the self-assembly behavior of these macromolecules was assessed using DLS (Fig. 2b). The DLS intensity distributions of both dendrons were monomodal with low polydispersity (PDI) values of 0.09 and 0.25 for BLM-Dendron and Ref-Dendron respectively, indicating narrow/moderate particle size distribution. Meanwhile, the average hydrodynamic diameter (*D*_H-avg_) of these dendrons was in the 200 nm range and their critical micelle concentration (CMC) was approximately 16 to 32 μg mL^−1^ based on the DLS count rate method (Fig. S11, ESI†).^54,55^ TEM analysis was also performed to visualize the self-assembled nanostructures where spherical particle morphologies with average diameters of *ca.* 44 and 52 nm for BLM-Dendron and Ref-Dendron respectively, were observed under the microscope (Fig. 2c). The discrepancy in particle diameters between DLS and TEM measurements is common and is most likely attributed to the fact that the dendrons were in a solvated state during DLS analysis (hence the *D*_H-avg_ includes the surrounding solvent layer) whereas with TEM, the dendrons were measured in a dried state. Based on the results of both DLS and TEM, the dendrons do indeed self-assemble in water as uniform nanoparticles. Besides investigating the self-assembly behavior, zeta potential measurements were also conducted on the dendrons. The zeta potential values obtained for BLM-Dendron and Ref-Dendron were −42 and 52 mV respectively, which are in line with colloidally stable anionic and cationic compounds.

**Fig. 3:**
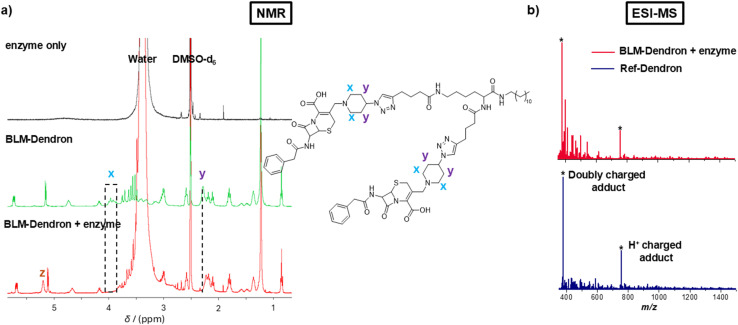
Uncaging investigation of BLM-Dendron. (a) NMR spectra depicting the changes to the resonances that correspond to the key functional groups of BLM-Dendron after incubation with penicillinase enzyme for 10 min. (b) ESI-MS spectra comparing Ref-Dendron and the enzyme-induced uncaged BLM-Dendron.

The uncaging efficiency of BLM-Dendron in the presence of penicillinase was investigated using ^1^H NMR spectroscopic analysis by following the resonances corresponding to key functional groups (Fig. 3a). This experiment had to be done in mostly deuterated DMSO solvent instead of water because of the low solubility of BLM-Dendron at the sufficient concentration required for reliable NMR spectroscopic analysis. Although not ideal since the biological assays were performed in water-based cell culture media, the data from the uncaging kinetics of BLM-Dendron in DMSO is still very useful in providing an indication on the uncaging process under biological conditions. As observed in Fig. 3a, the resonances of the alpha protons *x* and the methine protons *y* of BLM-Dendron shift when incubated with penicillinase for 10 min (at a concentration of 1 U per 0.35 mM of dendron). Specifically, the *x* protons shifted from *δ*_H_ 3.95 to 3.36 and 3.07 ppm (hidden by the large water peak) whereas the *y* protons shifted from *δ*_H_ 2.28 to 2.22 ppm. Furthermore, the hydrolysis of BLM-Dendron resulted in the appearance of a new peak *z* at *δ*_H_ 5.23 ppm that most likely corresponds to the ring-opened and released cephalosporin adduct, which agrees with the same observation made in another study that described the hydrolysis of a cephalosporin antibiotic with a metal–organic framework nanozyme.^56^ Identical spectra were acquired even at longer incubation times of up to 24 h, suggesting that the uncaging reaction was complete within 10 min (Fig. S12, ESI†). The rapidness at which the cephalosporin unit falls off in the presence of the enzyme is not entirely surprising as a previous study has observed a similar time scale in liberating nitric oxide from a cephalosporin family β-lactam prodrug using the same enzyme.^50^ Such high uncaging efficiency is desirable where precise burst activation of antimicrobial agents is required to immediately halt bacteria growth and stop the spread of infection. It is worth noting that the large water signal in the BLM-Dendron plus enzyme spectrum originated from the water used to prepare the enzyme stock solution, while the NMR spectra of the enzyme stock solution in deuterated DMSO did not produce any appreciable signal due to the very low enzyme concentration. Therefore, peak interference arising from the enzyme alone was ruled out as *a* factor.

The uncaging process was further confirmed using ESI-MS analysis. Specifically, ESI-MS was used to confirm the formation of BLM-Dendron (Fig. S13, ESI†) and to detect the generated cationic adduct following the uncaging process with penicillinase (Fig. 3b). After reacting BLM-Dendron with penicillinase, the occurrence of singly (377.8 Da) and doubly charged (754.6 Da) H^+^ cationic adducts as the dominant peaks in the mass spectra matched well with that of Ref-Dendron. This corroborated with the above NMR spectroscopy data showing that the uncaging reaction proceeds to completion. It should be noted that attempts were made to track the uncaging reaction *via* liquid chromatography analysis, but this was unsuccessful and complicated by the tendency of the dendrons to form self-assemblies, which made it difficult to differentiate between one another.

**Fig. 4:**
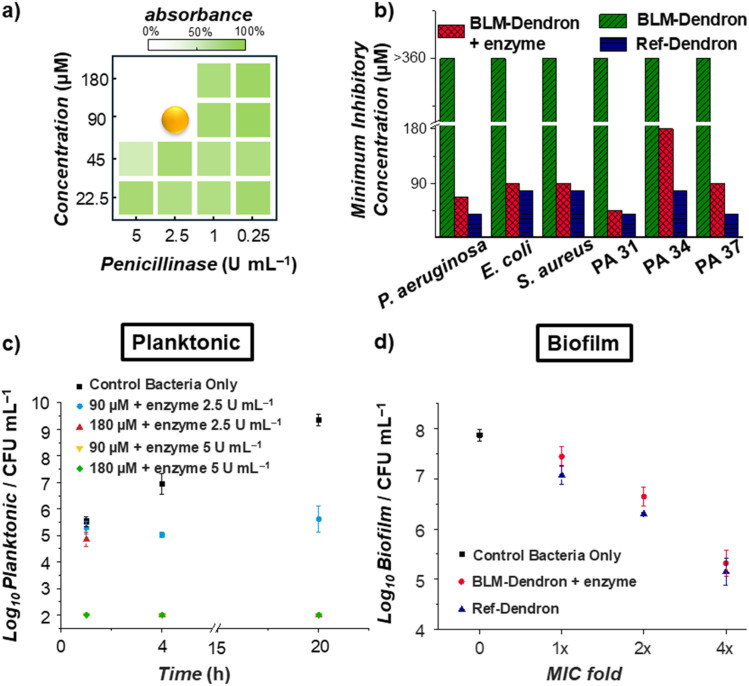
Antimicrobial performance of BLM-Dendron. (a) Simple minimum inhibitory concentration (MIC) checkerboard assay to determine the least amount of penicillinase enzyme required to activate BLM-Dendron to inhibit the growth of *P. aeruginosa* ATCC 27853. (b) The MIC values of BLM-Dendron in the presence and absence of penicillinase, and Ref-Dendron against *P. aeruginosa* ATCC 27853, *E. coli* K12, *S. aureus* ATCC 29213, and multidrug-resistant strains of *P. aeruginosa* PA31, PA34, and PA37. (c) Bactericidal time–kill activity of BLM-Dendron against planktonic *P. aeruginosa* ATCC 27853 cells in Mueller Hinton broth as determined *via* colony-forming unit (CFU) analysis. (d) Bactericidal activity of BLM-Dendron in comparison to Ref-Dendron at different concentrations against young biofilms of *P. aeruginosa* ATCC 27853 as determined by CFU analysis.

Next, the antimicrobial performance of BLM-Dendron was ascertained in terms of its bacteriostatic and bactericidal activities. Firstly, it was important to determine the optimal amount of penicillinase required to activate the dendron and for this, a simple minimum inhibitory concentration (MIC) checkerboard assay was performed where *P. aeruginosa* was challenged with different amounts of dendron and penicillinase to identify the minimum concentration pairing required to inhibit bacteria growth (Fig. 4a). Based on these preliminary results, the minimum amount of penicillinase needed to sufficiently uncage 90 μM BLM-Dendron (*i.e.* 128 μg mL^−1^) to induce antimicrobial activity was found to be 2.5 U mL^−1^, which translates to about 0.8 to 1.6 μg mL^−1^. To ensure there was sufficient enzyme to activate BLM-Dendron, 5 U mL^−1^ of penicillinase was employed in all subsequent antimicrobial assays, which was the same concentration used for the uncaging experiments (*vide supra*). The enzyme alone did not exhibit any bacteriostatic effects even up to 10 U mL^−1^.

The bacteriostatic activity of BLM-Dendron in the presence and absence of penicillinase was determined against wild-type *P. aeruginosa*, *Escherichia coli*, and *Staphylococcus aureus*, and several MDR *P. aeruginosa* (PA31, PA34 and PA37) strains^57^ (Fig. 4b). Without the addition of penicillinase, BLM-Dendron alone did not inhibit the growth of any of the bacteria even at the maximum tested concentration of 360 μM (*i.e.* 512 μg mL^−1^) since the cationic groups were still in their caged form. The same results were observed when a non-activating enzyme like galactosidase was used, indicating that the uncaging reaction was highly specific. In the presence of penicillinase, BLM-Dendron was uncaged *in situ* and displayed MIC values of 90 μM against the wild-type strains. These results are comparable to those of Ref-Dendron and suggested that the uncaged dendron could attain a similar level of antimicrobial potency as the unmodified original AMP mimic. Against MDR PA34 and PA37, the MIC values of the uncaged BLM-Dendron were twice as high as those of Ref-Dendron, whereas both dendrons showed near identical activity against MDR PA31. This suggests that certain MDR strains might affect the uncaging of BLM-Dendron, possibly due to the type of resistance genes present. A thorough investigation is needed to fully unravel this, which is beyond the scope of the current study.

It is worthwhile noting that the released and ring-opened cephalosporin adduct is highly unlikely in contributing to the observed antimicrobial activity of BLM-Dendron plus penicillinase samples. To support this claim, control experiments were conducted where Ref-Dendron was doped with imipenem, which is a β-lactam antibiotic, at 2 μg mL^−1^ (1 × MIC) along with penicillinase, and subjected to MIC assay against *P. aeruginosa*. This trio of components that include Ref-Dendron, imipenem and penicillinase resemble the identity of BLM-Dendron plus penicillinase sample. Should the ring-opened imipenem produce any antimicrobial effect, the control trio sample should yield lower and stronger MIC value than Ref-Dendron alone. However, this was not the case as both the control and Ref-Dendron exhibited identical MIC against *P. aeruginosa* and thus strongly implies that the observed antimicrobial activity of BLM-Dendron in the presence of the enzyme was indeed derived solely from the uncaged cationic dendron.

Additional experiments were performed to determine the killing efficiency of BLM-Dendron against wild-type *P. aeruginosa* in the presence of penicillinase. Firstly, against planktonic cells, 90 or 180 μM of the dendron (*i.e.* at 1 × MIC and 2 × MIC, respectively) was mixed with either 2.5 or 5 U mL^−1^ of penicillinase and *ca.* 5 × 10^5^ colony-forming unit per mL (CFU mL^−1^) of bacterial cells like in a typical setup for MIC assay, and left to incubate at 37 °C for 20 h. Samples were aliquoted at different time points to determine the amount of viable bacterial cells remaining at each juncture (Fig. 4c). At 1 h, samples with only 2.5 U mL^−1^ of enzyme did not yield any appreciable reduction in bacterial counts compared to the negative control (bacteria only sample) whereas the dendron completely eliminated all the bacteria (within the detection limit of 2 log_10_ in CFU mL^−1^) in the presence of 5 U mL^−1^ of penicillinase. At the 4 h timepoint, all samples showed no detectable bacteria except for the lowest combination pairing of 90 μM dendron plus 2.5 U mL^−1^ of penicillinase, which had a similar number of bacteria remaining as it did at the beginning of the experiment. This trend continued at the 20 h mark. Evidently, BLM-Dendron exhibited strong bactericidal effects with ≥99.9% killing efficiency within a short time frame when sufficient penicillinase (5 U mL^−1^) was available, which would not be possible unless the opening of the β-lactam ring and self-immolative process were rapid.

An important point to note is that the antimicrobial assays above were performed in nutrient rich culture media that resemble the high protein and salt contents similar to biological conditions, and yet the uncaging reaction and conferment of antimicrobial activity proceeded efficiently *in situ*, unlike other enzyme stimuli responsive systems which generally showed reduced activity in biological media, including an earlier system reported by us.^49^ Previously, we have described the concept of antimicrobial dendrons based on AUS using β-galactosidase enzyme as the trigger. The uncaging reaction effectively proceeded in water or phosphate buffered saline (PBS) but failed in culture media. This contrast in performance thus highlights the advantage of using a more labile caging agent such as the strained, square-like structure in β-lactam motifs for antimicrobial prodrug development.

By definition, biofilms are a network of cells that are harder to eradicate compared to planktonic cells because of the protection offered by the matrix of extracellular substances. Given that BLM-Dendron demonstrated good efficacy against planktonic cells, we subsequently investigated its ability to combat bacteria biofilms. Specifically, young biofilms of wild-type *P. aeruginosa* were grown in M9 minimal medium for 6 h according to our previous protocol^53^ and were later exposed to the dendrons for 1 h at different concentrations (*i.e.* at 1 × MIC, 2 × MIC and 4 × MIC), followed by CFU analysis to determine the amount of viable biofilm cells remaining (Fig. 4d). The antibiofilm activity of BLM-Dendron in the presence of 5 U mL^−1^ penicillinase was indistinguishable to Ref-Dendron, further indicating that the uncaged dendron is fully active. As predicted, biofilms are indeed harder to eradicate and approximately only 1.5 log_10_ reduction in CFU mL^−1^ compared to the untreated control was achieved even at 2 × MIC concentration. However, better elimination efficiency was observed at 4 × MIC concentration (2.5 log_10_ reduction in CFU mL^−1^). The need for higher doses to eradicate biofilm cells compared to planktonic cells is consistent with other AMP mimics reported in literature.^58–60^

**Fig. 5:**
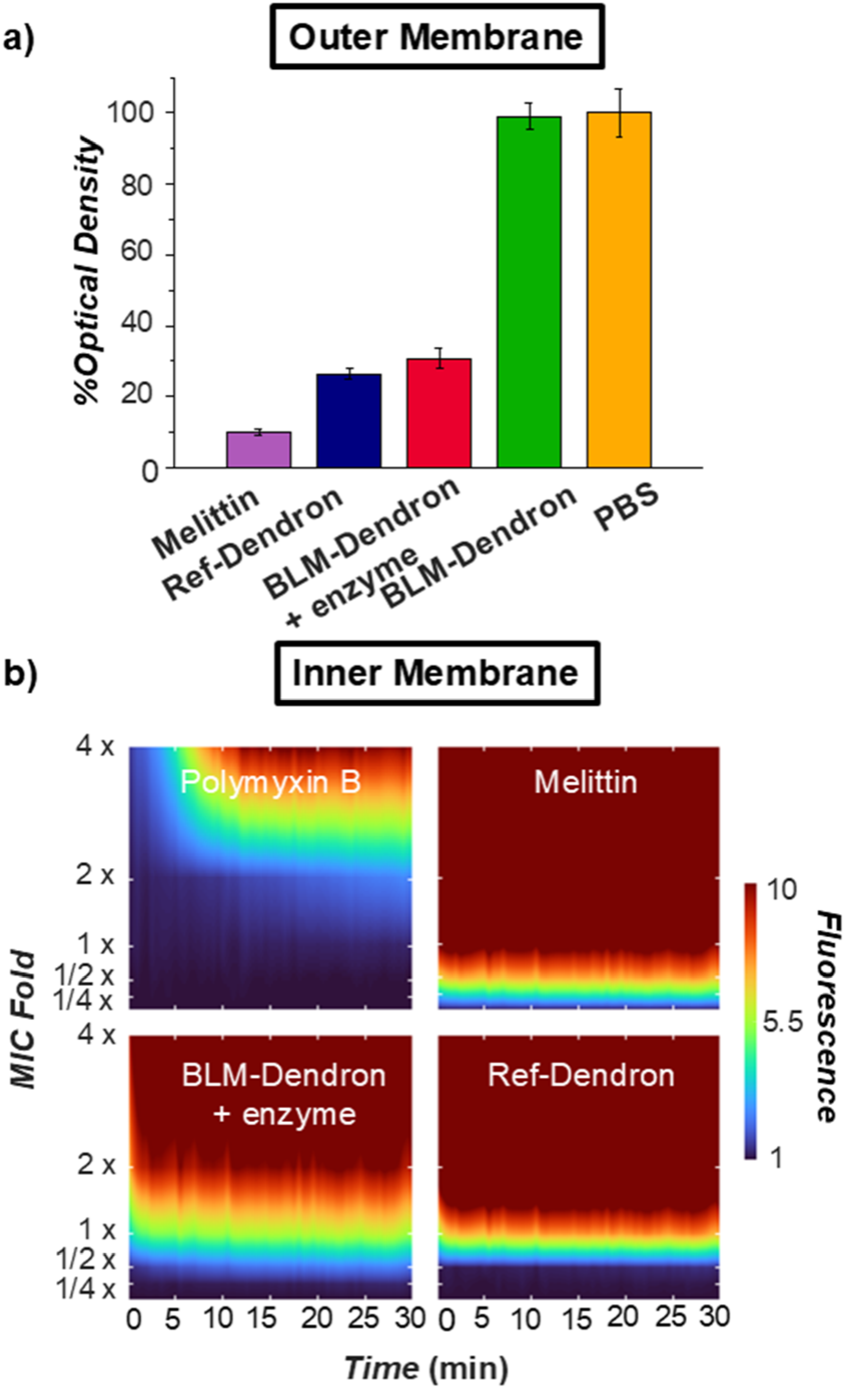
Bacteria membrane activity imparted by BLM-Dendron. (a) The ability to sensitize the outer membrane of *P. aeruginosa* ATCC 27853 at 1 × MIC to the lytic action of sodium deoxycholate, as indicated by the percentage optical density (at 485 nm) of the final treated samples relative to the negative control (*i.e.*, PBS containing sodium deoxycholate). (b) Inner membrane disruption study on *P. aeruginosa* ATCC 27853 where the membrane permeability variation, as quantified by a dimensionless constant (the fluorescence fold change between the treatment group and the negative control group, measured at excitation and emission wavelengths of 544 and 622 nm, respectively), was illustrated as a heat map with the resolution of time and concentration based on linear regression and plotted using MATLAB. Melittin and polymyxin B were included as the positive control, and their MIC values were 32 and 2 μg mL^−1^, respectively.

Considering that Ref-Dendron disrupts the bacterial cell membrane because of its cationic amphipathic structure, two membrane mechanism experiments were conducted to verify the ability of the uncaged BLM-Dendron to act on the outer and inner membranes of wild-type *P. aeruginosa* (Fig. 5). Firstly, the dendrons were checked for their interaction with the outer membrane where the bacterial cells were briefly sensitized at 1 × MIC concentration for 10 min prior to a second incubation with the anionic surfactant sodium deoxycholate for a further 10 min. If the dendrons compromise the outer membrane integrity, the bacteria cells would then lyse upon further treatment with sodium deoxycholate and this could be measured in terms of the change in optical density (Fig. 5a). Ref-Dendron and the AMP melittin (positive control) resulted in >70% reduction in optical density compared to the PBS-treated negative control, whereas the caged BLM-Dendron did not result in any reportable cell lysis. In the presence of penicillinase, the uncaged BLM-Dendron led to 70% reduction in optical density, which was comparable to Ref-Dendron.

The ability of the dendrons to permeate the inner membrane was assessed using propidium iodide (PI) assay (Fig. 5b). PI does not traverse across intact cell membrane and will only emit red light if the dye could penetrate weakened membrane walls and intercalate with nucleic acids. Hence, the extent of inner membrane permeability is correlated to the red fluorescence intensity of PI. Fig. 5b displays the heatmap plots of the dendrons as well as AMPs melittin and polymyxin B for comparison. Melittin was found to have the strongest effect on cell lysis, completely permeabilizing the inner membrane in less than a minute at 0.5 × MIC, followed closely by Ref-Dendron at 1 × MIC in 1 min, and BLM-Dendron at 2 × MIC also in 1 min with added penicillinase. Interestingly, polymyxin B was the weakest and required 4 × MIC and nearly 10 min to cause maximum disruption of the inner membrane of *P. aeruginosa*. Taken together, the uncaged BLM-Dendron is certainly membrane active with similar levels of disruption compared to Ref-Dendron.

**Fig. 6:**
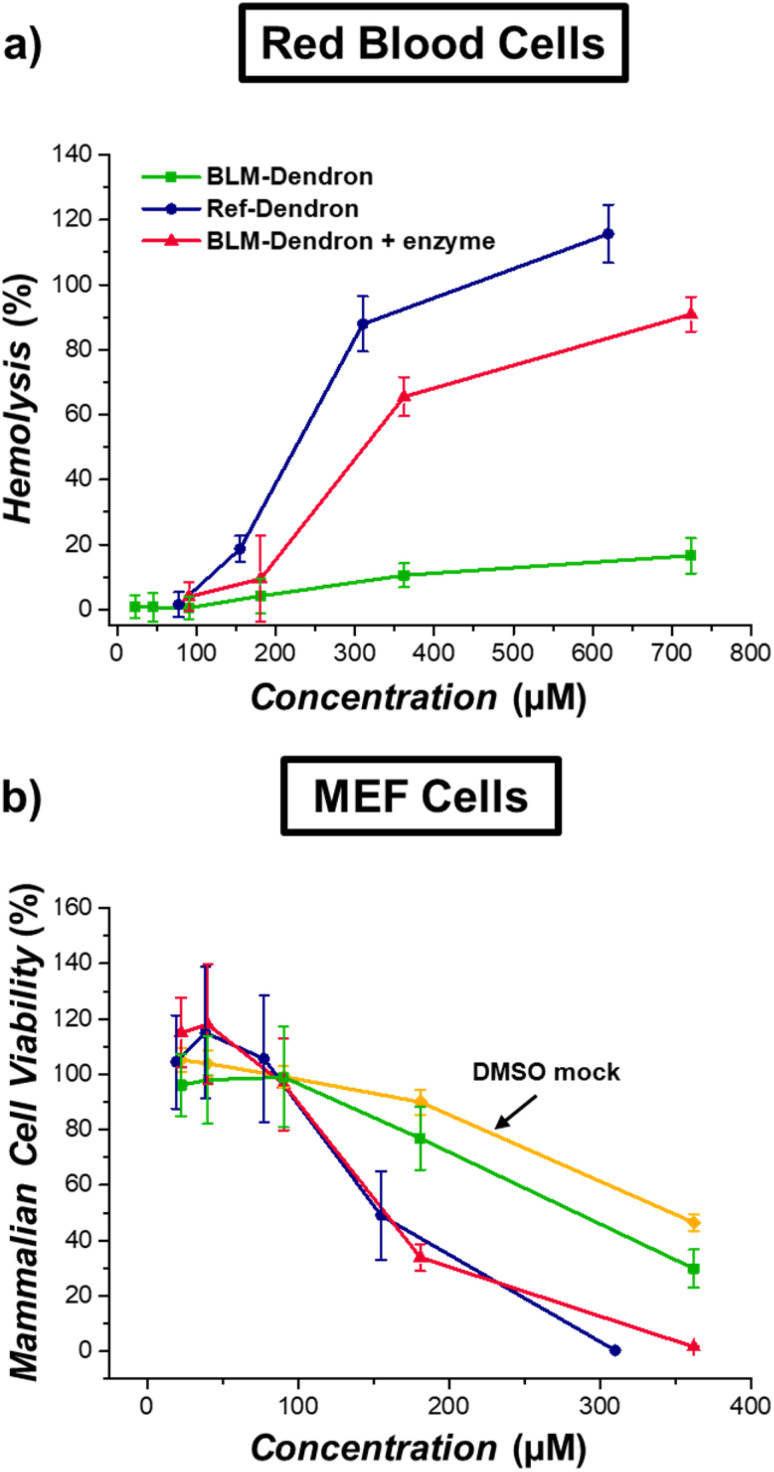
Biocompatibility of BLM-Dendron. (a) Extent of hemolysis on sheep red blood cells after incubation with different concentrations of BLM-Dendron in the presence and absence of penicillinase enzyme, and Ref-Dendron for 1 h at 37 °C. (b) Cell viability curves of mouse embryonic fibroblast (MEF) cells as a function of compound concentration after incubation at 37 °C for 24 h.

Finally, the biocompatibility of BLM-Dendron was determined to ensure that the caging of the amine groups would lead to lower toxicity. This was assessed using sheep red blood cells (RBCs) and murine embryonic fibroblast (MEF) cells *via* hemolytic and cell viability studies, respectively (Fig. 6). In the absence of enzyme trigger, BLM-Dendron had very low hemolytic activity (<20%) even at the highest tested concentration of 720 μM. In contrast, Ref-Dendron lysed 50% of RBCs (*i.e.*, HC_50_ value) at 200 μM while uncaged BLM-Dendron has an HC_50_ value of 300 μM. The caged amines undeniably resulted in significant improvement in terms of compatibility with RBCs compared to the uncaged counterparts. Furthermore, when comparing in terms of selectivity (*i.e.*, ratio of HC_50_ to MIC), BLM-Dendron has a selectivity of > 8 while Ref-Dendron and the uncaged BLM-Dendron have selectivity values of 5.7 and 3.3, respectively. While it could be argued that BLM-Dendron will inadvertently cause toxicity to surrounding mammalian cells when activated, the toxicity would most likely be localized to infection sites and not during circulation if the compound was to be administered as a therapeutic, given the excellent hemocompatibility of the amine-caged BLM-Dendron.

The cytotoxicity of the dendrons towards MEF cells was determined based on the metabolic activity after 24 h incubation and compared in terms of the IC_50_ value, which is defined as the half-maximal concentration that reduces the cell viability by half (Fig. 6b). Likewise in the hemolytic study, both Ref-Dendron and uncaged BLM-Dendron had similar levels of toxicity with IC_50_ values of *ca.* 150 μM. On the other hand, the IC_50_ of BLM-Dendron appeared to be double that of the uncaged form, although we suspect that its true value would be higher if not for the increased content of DMSO solvent at higher compound concentrations. It is worth noting again that DMSO was used to prepare stock solution of BLM-Dendron for biological testing at 20 mg mL^−1^. The viability of MEF cells was clearly affected by DMSO at higher concentrations as observed in Fig. 6b where the DMSO mock control sample, which contained the same amount of solvent as in the BLM-Dendron sample but without the dendron, showed similar IC_50_ value to the BLM-Dendron sample. This strongly suggests that the toxicity profile witnessed for BLM-Dendron was derived from the solvent and not necessarily due to the compound itself. Regardless, even in the worst-case scenario, BLM-Dendron is still at least twice as biocompatible than the exposed cationic dendron.

## Conclusion

In summary, we developed a new antimicrobial peptide mimic prodrug BLM-Dendron based on the amine uncaging strategy (AUS) where the amine groups of a Y-shaped cationic amphipathic dendron are caged with a cleavable cephalosporin motif. The motivation behind this study was to judiciously mask the cationic groups, which are responsible for causing toxicity, to improve the biocompatibility of the therapeutic agent, whilst enabling precise antimicrobial activation using a specific trigger. Using penicillinase as the trigger, which is a β-lactamase enzyme produced exclusively by bacteria, the amine groups of BLM-Dendron could be uncaged within 10 min to confer on-demand antimicrobial activity against Gram-negative and Gram-positive pathogens including (multidrug-resistant) *P. aeruginosa*, *E. coli* and *S. aureus*. The uncaged BLM-Dendron exhibited bacteriostatic and bactericidal activities and was also effective in killing biofilm cells. Like other cationic amphipathic antimicrobials, once uncaged, the dendron was found to exert its activity by disrupting the bacteria cell wall membranes. Crucially, BLM-Dendron had superior hemocompatibility and lower toxicity towards fibroblast cells compared to the exposed cationic version. This study thus demonstrates the advantage of using AUS to convert potentially toxic membrane-active antimicrobial agents into more biocompatible drug molecules with targeted activation capabilities, which would be highly desirable for clinical applications.

## Data availability

The data supporting this article have been included as part of the ESI.†

## Author contributions

H. L., Z. S., K. H., R. P. K., and S. K. M. performed or assisted with the experiments and analysis and were specifically responsible for investigation, methodology, formal analysis, and validation. J. X. and M. W. helped with supervision. H. L. was also responsible for data curation, conceptualization, visualization, project administration, and writing the original draft. E. H. H. W. was responsible for conceptualization, resources, funding acquisition, supervision, project administration, and writing the original draft. All authors were involved with reviewing and editing the manuscript.

## Conflicts of interest

There are no conflicts to declare.

## Supplementary Material

SC-OLF-D5SC02412A-s001

## References

[cit1] Ikhimiukor O. O., Odih E. E., Donado-Godoy P., Okeke I. N. (2022). A bottom-up view of antimicrobial resistance transmission in developing countries. Nat. Microbiol..

[cit2] Nathan C. (2020). Resisting antimicrobial resistance. Nat. Rev. Microbiol..

[cit3] New report calls for urgent action to avert antimicrobial resistance crisis, 20no%20action%20is%20taken,2008%2D2009%20global%20financial%20crisis, (accessed April 29, 2019)

[cit4] Antimicrobial resistance, https://www.who.int/news-room/fact-sheets/detail/antimicrobial-resistance, (accessed September 19, 2024)

[cit5] Lewis J. M., Mphasa M., Banda R., Beale M. A., Heinz E., Mallewa J., Jewell C., Faragher B., Thomson N. R., Feasey N. A. (2022). Colonization dynamics of extended-spectrum beta-lactamase-producing Enterobacterales in the gut of Malawian adults. Nat. Microbiol..

[cit6] Bush K., Bradford P. A. (2019). Interplay between β-lactamases and new β-lactamase inhibitors. Nat. Rev. Microbiol..

[cit7] Nantongo M., Nguyen D. C., Bethel C. R., Taracila M. A., Li Q., Dousa K. M., Shin E., Kurz S. G., Nguyen L., Kreiswirth B. N., Boom W. H., Plummer M. S., Bonomo R. A. (2024). Durlobactam, a Diazabicyclooctane β-Lactamase Inhibitor, Inhibits BlaC and Peptidoglycan Transpeptidases of. ACS Infect. Dis..

[cit8] Schechter L. M., Creely D. P., Garner C. D., Shortridge D., Nguyen H., Chen L., Hanson B. M., Sodergren E., Weinstock G. M., Dunne W. M., van Belkum A., Leopold S. R. (2018). Extensive Gene Amplification as a Mechanism for Piperacillin-Tazobactam Resistance in Escherichia coli. mBio.

[cit9] Shields R. K., Chen L., Cheng S. J., Chavda K. D., Press E. G., Snyder A., Pandey R., Doi Y., Kreiswirth B. N., Nguyen M. H., Clancy C. J. (2017). Emergence of Ceftazidime-Avibactam Resistance Due to Plasmid-Borne bla_KPC-3_ Mutations during Treatment of Carbapenem-Resistant Klebsiella pneumoniae Infections. Antimicrob. Agents Chemother..

[cit10] Livermore D. M., Mushtaq S., Warner M., Vickers A., Woodford N. (2017). In vitro activity of cefepime/zidebactam (WCK 5222) against Gram-negative bacteria. J. Antimicrob. Chemother..

[cit11] Pilmis B., Jullien V., Tabah A., Zahar J. R., Brun-Buisson C. (2017). Piperacillin-tazobactam as alternative to carbapenems for ICU patients. Ann. Intensive Care.

[cit12] Wu Y. M., Chen K., Wang J. Z., Chen M. Z., Chen Y., She Y. R., Yan Z., Liu R. H. (2023). Host defense peptide mimicking antimicrobial amino acid polymers and beyond: Design, synthesis and biomedical applications. Prog. Polym. Sci..

[cit13] Zhang H. D., Chen Q., Xie J. Y., Cong Z. H., Cao C. T., Zhang W. J., Zhang D. H., Chen S., Gu J. W., Deng S., Qiao Z. Q., Zhang X. Y., Li M. Q., Lu Z. Y., Liu R. H. (2023). Switching from membrane disrupting to membrane crossing, an effective strategy in designing antibacterial polypeptide. Sci. Adv..

[cit14] Magana M., Pushpanathan M., Santos A. L., Leanse L., Fernandez M., Ioannidis A., Giulianotti M. A., Apidianakis Y., Bradfute S., Ferguson A. L., Cherkasov A., Seleem M. N., Pinilla C., de la Fuente-Nunez C., Lazaridis T., Dai T. H., Houghten R. A., Hancock R. E. W., Tegos G. P. (2020). The value of antimicrobial peptides in the age of resistance. Lancet Infect. Dis..

[cit15] Guo W., Wang Y. J., Wan P. Q., Wang H., Chen L., Zhang S. K., Xiao C. S., Chen X. S. (2022). Cationic amphiphilic dendrons with effective antibacterial performance. J. Mater. Chem. B.

[cit16] Shabani S., Hadjigol S., Li W. Y., Si Z. Y., Pranantyo D., Chan-Park M. B., O'Brien-Simpson N. M., Qiao G. G. (2024). Synthetic peptide branched polymers for antibacterial and biomedical applications. Nat. Rev. Bioeng..

[cit17] Song J. Y., Cortez-Jugo C., Shirbin S. J., Lin Z. X., Pan S. J., Qiao G. G., Caruso F. (2022). Immobilization and Intracellular Delivery of Structurally Nanoengineered Antimicrobial Peptide Polymers Using Polyphenol-Based Capsules. Adv. Funct. Mater..

[cit18] Shirbin S. J., Insua I., Holden J. A., Lenzo J. C., Reynolds E. C., O'Brien-Simpson N. M., Qiao G. G. (2018). Architectural Effects of Star-Shaped “Structurally Nanoengineered Antimicrobial Peptide Polymers” (SNAPPs) on Their Biological Activity. Adv. Healthc. Mater..

[cit19] Maset R. G., Hapeshi A., Hall S., Dalgliesh R. M., Harrison F., Perrier S. (2022). Evaluation of the Antimicrobial Activity in Host-Mimicking Media and Toxicity of Antimicrobial Polymers as Functional Mimics of AMPs. ACS Appl. Mater. Inter..

[cit20] Takahashi H., Caputo G. A., Kuroda K. (2021). Amphiphilic polymer therapeutics: an alternative platform in the fight against antibiotic resistant bacteria. Biomater. Sci..

[cit21] Li W. Y., Separovic F., O'Brien-Simpson N. M., Wade J. D. (2021). Chemically modified and conjugated antimicrobial peptides against superbugs. Chem. Soc. Rev..

[cit22] Barman S., Konai M. M., Samaddar S., Haldar J. (2019). Amino Acid Conjugated Polymers: Antibacterial Agents Effective against Drug-Resistant with No Detectable Resistance. ACS Appl Mater. Inter..

[cit23] Gao J. Y., Wang M. Z., Wang F. Y. K., Du J. Z. (2016). Synthesis and Mechanism Insight of a Peptide-Grafted Hyperbranched Polymer Nanosheet with Weak Positive Charges but Excellent Intrinsically Antibacterial Efficacy. Biomacromolecules.

[cit24] Wang T., Teng R. X., Wu M. J., Ge Z. H., Liu Y. P., Yang B., Li C., Fan Z., Du J. Z. (2024). A Polypeptosome Spray To Heal Antibiotic-Resistant Bacteria-Infected Wound by Photocatalysis-Induced Metabolism-Interference. ACS Nano.

[cit25] Zhou Q., Si Z. Y., Wang K., Li K. P., Hong W. L., Zhang Y. Z., Li P. (2022). Enzyme-triggered smart antimicrobial drug release systems against bacterial infections. J. Contr. Release..

[cit26] Barman S., Kurnaz L. B., Yang X. M., Nagarkatti M., Nagarkatti P., Decho A. W., Tang C. B. (2023). Facially Amphiphilic Bile Acid-Functionalized Antimicrobials: Combating Pathogenic Bacteria, Fungi, and Their Biofilms. ACS Infect. Dis..

[cit27] Phuong P. T., Oliver S., He J. C., Wong E. H. H., Mathers R. T., Boyer C. (2020). Effect of Hydrophobic Groups on Antimicrobial and Hemolytic Activity: Developing a Predictive Tool for Ternary Antimicrobial
Polymers. Biomacromolecules.

[cit28] Qian Y. X., Deng S., Cong Z. H., Zhang H. D., Lu Z. Y., Shao N., Bhatti S. A., Zhou C., Cheng J. G., Gellman S. H., Liu R. H. (2022). Secondary Amine Pendant β-Peptide Polymers Displaying Potent Antibacterial Activity and Promising Therapeutic Potential in Treating MRSA-Induced Wound Infections and Keratitis. J. Am. Chem. Soc..

[cit29] Sun J., Li M., Zhang B., Chen X. S. (2021). High Antibacterial Activity and Selectivity of the Versatile Polysulfoniums that Combat Drug Resistance. Adv. Mater..

[cit30] Zhao H. M., Zhong L. L., Yang C. A., Tang N., He Y. W., He W., Zhao Z. H., Wu C. B., Yuan P. Y., Yang Y. Y., Tian G. B., Ding X. (2023). Antibiotic-Polymer Self-Assembled Nanocomplex to Reverse Phenotypic Resistance of Bacteria toward Last-Resort Antibiotic Colistin. ACS Nano.

[cit31] Tan J. S., Fang Y. H., Yang C., Tay J., Tan N., Krishnan N. B., Chua B. L., Zhao Y. L., Chen Y. B., Hedrick J. L., Yang Y. Y. (2023). pH-Responsive Polymeric Micelle Dynamic Complexes for Selective Killing of. Biomacromolecules.

[cit32] Ding X., Yang C., Moreira W., Yuan P. Y., Periaswamy B., de Sessions P. F., Zhao H. M., Tan J. R., Lee A., Ong K. X., Park N., Liang Z. C., Hedrick J. L., Yang Y. Y. (2020). A Macromolecule Reversing Antibiotic Resistance Phenotype and Repurposing Drugs as Potent Antibiotics. Adv. Sci..

[cit33] Zhang B., Lu D. R., Wang D. B. R., Kok Z. Y., Chan-Park M. B., Duan H. W. (2024). Enzyme-Responsive Polyion Complex Nanoparticles of Cationic Antimicrobials for Activatable Antibacterial Therapy. Adv. Funct. Mater..

[cit34] Kanwal S., Aziz U. B., Quaas E., Achazi K., Klinger D. (2025). Sulfonium-based polymethacrylamides for antimicrobial use: influence of the structure and composition. Biomater. Sci..

[cit35] Thaker H. D., Som A., Ayaz F., Lui D. H., Pan W. X., Scott R. W., Anguita J., Tew G. N. (2012). Synthetic Mimics of Antimicrobial Peptides with Immunomodulatory Responses. J. Am. Chem. Soc..

[cit36] Lehnen A. C., Kogikoski S. J. r., Stensitzki T., AlSawaf A., Bapolisi A. M., Wolff M., De Breuck J., Müller-Werkmeister H. M., Chiantia S., Bald I., Leiske M. N., Hartlieb M. (2024). Anisotropy in Antimicrobial Bottle Brush Copolymers and Its Influence on Biological Activity. Adv. Funct. Mater..

[cit37] Chen A., Karanastasis A., Casey K. R., Necelis M., Carone B. R., Caputo G. A., Palermo E. F. (2020). Cationic Molecular Umbrellas as Antibacterial Agents with Remarkable Cell-Type Selectivity. ACS Appl. Mater. Inter..

[cit38] Rahman M. A., Bam M., Luat E., Jui M. S., Ganewatta M. S., Shokfai T., Nagarkatti M., Decho A. W., Tang C. B. (2018). Macromolecular-clustered facial amphiphilic antimicrobials. Nat. Commun..

[cit39] Li W. Y., Hadjigol S., Mazo A. R., Holden J., Lenzo J., Shirbin S. J., Barlow A., Shabani S., Huang T., Reynolds E. C., Qiao G. G., O'Brien-Simpson N. M. (2022). Star-Peptide Polymers are Multi-Drug-Resistant Gram-Positive Bacteria Killers. ACS Appl. Mater. Inter..

[cit40] Zhou M., Liu L. Q., Cong Z. H., Jiang W. N., Xiao X. M., Xie J. Y., Luo Z. J., Chen S., Wu Y. M., Xue X. Y., Shao N., Liu R. H. (2024). A dual-targeting antifungal is effective against multidrug-resistant human fungal pathogens. Nat. Microbiol..

[cit41] Lin B. C., Hung A. D., Singleton W., Darmawan K. K., Moses R., Yao B. C., Wu H. K., Barlow A., Marc-Antoine S., Sloan A. J., Hossain M. A., Wade J. D., Hong Y. N., O'Brien-Simpson N. M., Li W. Y. (2023). The effect of tailing lipidation on the bioactivity of antimicrobial peptides and their aggregation tendency. Aggregate.

[cit42] Shao Z. Y., Luo H., Nguyen T. H. Q., Wong E. H. H. (2024). Effects of Secondary Amine and Molecular Weight on the Biological Activities of Cationic Amphipathic Antimicrobial Macromolecules. Biomacromolecules.

[cit43] Shao Z. Y., Wulandari E., Lin R. C. Y., Xu J. T., Liang K., Wong E. H. H. (2022). Two plus One: Combination Therapy Tri-systems Involving Two Membrane-Disrupting Antimicrobial Macromolecules and Antibiotics. ACS Infect. Dis..

[cit44] Javadi H., Lehnen A., Hartlieb M. (2025). Bioinspired Cationic Antimicrobial Polymers. Angew. Chem., Int. Ed..

[cit45] Insua I., Liamas E., Zhang Z. Y., Peacock A. F. A., Krachler A. M., Fernandez-Trillo F. (2016). Enzyme-responsive polyion complex (PIC) nanoparticles for the targeted delivery of antimicrobial polymers. Polym. Chem..

[cit46] Ergene C., Palermo E. (2019). Self-immolative polymers with potent and selective antibacterial activity by hydrophilic side chain grafting. J. Mater. Chem. B.

[cit47] Zheng W., Anzaldua M., Arora A., Jiang Y. J., McIntyre K., Doerfert M., Winter T., Mishra A., Ma H. R., Liang H. J. (2020). Environmentally Benign Nanoantibiotics with a Built-in Deactivation Switch Responsive to Natural Habitats. Biomacromolecules.

[cit48] Shao Z. Y., Hakobyan K., Xu J. T., Chen R. X., Kumar N., Willcox M., Wong E. H. H. (2023). Photoinduced Unveiling of Cationic Amine: Toward Smart Photoresponsive Antimicrobial Polymers. ACS Appl. Polym. Mater..

[cit49] Shao Z. Y., Xu Y. D., Luo H., Hakobyan K., Zhang M. N., Xu J. T., Stenzel M. H., Wong E. H. H. (2024). Smart Galactosidase-Responsive Antimicrobial Dendron: Towards More Biocompatible Membrane-Disruptive Agents. Macromol. Rapid.

[cit50] Barraud N., Kardak B. G., Yepuri N. R., Howlin R. P., Webb J. S., Faust S. N., Kjelleberg S., Rice S. A., Kelso M. J. (2012). Cephalosporin-3′-diazeniumdiolates: Targeted NO-Donor Prodrugs for Dispersing Bacterial Biofilms. Angew. Chem., Int. Ed..

[cit51] Rineh A., Soren O., McEwan T., Ravikumar V., Poh W. H., Azamifar F., Naimi-Jamal M. R., Cheung C. Y., Elliott A. G., Zuegg J., Blaskovich M. A. T., Cooper M. A., Dolange V., Christodoulides M., Cook G. M., Rice S. A., Faust S. N., Webb J. S., Kelso M. J. (2020). Discovery of Cephalosporin-3′-Diazeniumdiolates That Show Dual Antibacterial and Antibiofilm Effects against Clinical Cystic Fibrosis Isolates and Efficacy in a Murine Respiratory Infection Model. ACS Infect. Dis..

[cit52] Li Y. M., Liu G. H., Wang X. R., Hu J. M., Liu S. Y. (2016). Enzyme-Responsive Polymeric Vesicles for Bacterial-Strain-Selective Delivery of Antimicrobial Agents. Angew. Chem., Int. Ed..

[cit53] Alkekhia D., LaRose C., Shukla A. (2022). β-Lactamase-Responsive Hydrogel Drug Delivery Platform for Bacteria-Triggered Cargo Release. ACS Appl. Mater. Inter..

[cit54] Noy J. M., Chen F., Akhter D. T., Houston Z. H., Fletcher N. L., Thurecht K. J., Stenzel M. H. (2020). Direct Comparison of Poly(ethylene glycol) and Phosphorylcholine Drug-Loaded Nanoparticles *In Vitro* and *In Vivo*. Biomacromolecules.

[cit55] Callari M., De Souza P. L., Rawal A., Stenzel M. H. (2017). The Effect of Drug Loading on Micelle Properties: Solid-State NMR as a Tool to Gain Structural Insight. Angew. Chem., Int. Ed..

[cit56] Escamilla P., Bartella L., Sanz-Navarro S., Percoco R. M., Di Donna L., Prejanò M., Marino T., Ferrando-Soria J., Armentano D., Leyva-Perez A., Pardo E. (2023). Degradation of Penicillinic Antibiotics and β-Lactamase Enzymatic Catalysis in a Biomimetic Zn-Based Metal-Organic Framework. Chem.–Eur. J..

[cit57] Subedi D., Kumarvijay A., Kohli G. S., Rice S. A., Willcox M. (2018). Comparative genomics of clinical strains of strains isolated from different geographic sites. Sci. Rep..

[cit58] Nguyen T. K., Lam S. J., Ho K. K. K., Kumar N., Qiao G. G., Egan S., Boyer C., Wong E. H. H. (2017). Rational Design of Single-Chain Polymeric Nanoparticles That Kill Planktonic and Biofilm Bacteria. ACS Infect. Dis..

[cit59] Liu Y., Busscher H. J., Zhao B. R., Li Y. F., Zhang Z. K., van der Mei H. C., Ren Y. J., Shi L. Q. (2016). Surface-Adaptive, Antimicrobially Loaded, Micellar Nanocarriers with Enhanced Penetration and Killing Efficiency in Staphylococcal Biofilms. ACS Nano.

[cit60] Kurnaz L. B., Barman S., Yang X. M., Fisher C., Outten F. W., Nagarkatti P., Nagarkatti M., Tang C. B. (2023). Facial amphiphilic naphthoic acid-derived antimicrobial polymers against multi-drug resistant gram-negative bacteria and biofilms. Biomaterials.

