# Supplementary material for: β-Lactamase-activated antimicrobial dendron via the amine uncaging strategy
Source: Chem Sci. 2025 May 12;16(23):10296–306. doi: 10.1039/d5sc02412a (PMC12079575; doi:10.1039/d5sc02412a)
Supplement: SC-016-D5SC02412A-s001 [file SC-016-D5SC02412A-s001.pdf]

Supplementary Information (SI) for Chemical Science.
This journal is © The Royal Society of Chemistry 2025

Supporting Information (SI)

β-Lactamase-Activated Antimicrobial Dendron via the Amine Uncaging Strategy

Hao Luo,[a] Zeyu Shao,[a] Karen Hakobyan,[a] Jiangtao Xu,[a] Rhiannon P. Kuchel,[b] Shyam Kumar Mishra,[c] Mark Willcox,[c] and Edgar H. H. Wong[a]*

[a]School of Chemical Engineering, University of New South Wales, Sydney, NSW 2052, Australia.

[b]Electron Microscope Unit, University of New South Wales, Sydney, NSW 2052, Australia.

[c]School of Optometry and Vision Science, University of New South Wales, Sydney, NSW 2052, Australia.

Email: edgar.wong@unsw.edu.au

EXPERIMENTAL SECTION

Materials

$N^{\alpha,\varepsilon}$-Bis-Boc-*L*-lysine *N*-hydroxysuccinimide ester (Chem-Inpex International, 99%, dodecylamine (Sigma-Aldrich, 98%), sodium iodide (Chem-Supply), sodium thiosulfate (Sigma-Aldrich), 5-hexynoic acid (Sigma-Aldrich, 97%), 1-ethyl-3-(3-dimethylaminopropyl)carbodiimide hydrochloride (Chem-Supply, >98%), trifluoroacetic acid (TFA, Chem-Supply), copper (I) iodide (Sigma-Aldrich, 98%), 7-phenylacetamido-3-chloromethyl-3-cephem-4-carboxylic acid-*p*-methoxybenzyl ester (AK Scientific, 97%), *N,N,N',N",N"*-pentamethyldiethylenetriamine (PMDETA) (Sigma-Aldrich, 99%), thioanisole (Sigma-Aldrich, 99%), hydrogen chloride solution (Sigma-Aldrich, 4 M in 1,4-dioxane), propidium iodide (Sigma-Aldrich, ≥94%), Triton X-100 (Sigma-Aldrich, laboratory grade), penicillinase from *Bacillus cereus* (Sigma-Aldrich, lyophilized powder, 1,500-3,000 units/mg protein) and sodium deoxycholate (Sigma-Aldrich, ≥97%) were used as received. Ethylenediaminetetraacetic acid (EDTA), sodium chloride (NaCl), magnesium sulfate ($MgSO_4$), sodium hydrogen carbonate ($NaHCO_3$), 1,4-dioxane, dimethylformamide, acetone, methanol, chloroform, ethyl acetate, hexane, and diethyl ether were obtained from Chem-Supply and used as received. Milli-Q water with a resistivity of >18.2 MΩ·cm was obtained from an in-line Millipore RiOs/Origin water purification system.

Chemical Characterizations

Nuclear magnetic resonance

All ^{1}H and ^{13}C nuclear magnetic resonance (NMR) spectroscopies were performed by a Bruker 300 or 400 MHz spectrometer using deuterated solvents (obtained from Cambridge Isotope Laboratories) as reference solvents and at a sample concentration of ca. 10–20 mg mL^{-1}.

Gel permeation chromatography

Gel permeation chromatography (GPC) was carried out on a Shimadzu liquid chromatography system equipped with an SIL-20A auto-injector, a Polymer Laboratories 5.0 μm bead-size guard column (50 × 7.5 mm^2) followed by three linear PL (Styragel) columns (10^5, 10^4 and 10^3 Å) and an RID-10A differential refractive-index (RI) detector operating at 50 °C. Dimethylacetamide (DMAc) (containing 0.03% w/v LiBr and 0.05% w/v 2,6-dibutyl-4-methylphenol (BHT)) was used as the eluent at a flow rate of 1 mL min^{-1}. The system was calibrated with poly(methyl methacrylate) (PMMA) standards with molecular weights from 200 to 10^6 g mol^{-1}.

Dynamic light scattering and zeta potential

Dynamic light scattering and zeta-potential measurements were performed using a Malvern Zetasizer Ultra–Red (Malvern Panalytical). The compounds were analysed at 25 °C in deionized water, where the sample solution was filtered through a 0.45 μm pore size filter and pipetted into a folded capillary cell (DTS1070, Malvern Panalytical). The critical micelle concentration (CMC) was determined by the mean count rate of the scattered light against serially diluted samples in which the measurement parameters of DLS (e.g., the attenuator and cell position) were fixed.[1, 2]

Electrospray ionization mass spectrometry

Electrospray ionization mass spectrometry (ESI–MS) was performed on a Thermo LTQ Orbitrap XL equipped with an ESI source. The *m/z* axis was calibrated to within 2 ppm of accuracy using a solution of Ultramark 1621. The ESI ionization parameters were as follow: spray potential 1.5 V, capillary temperature 200 ^{o}C, capillary potential 33 V, and tube lens potential 100 V.

Transmission electron microscopy

TEM imaging was performed using a Thermo Fischer Scientific Talos 120C microscope operating at an accelerating voltage of 120 keV and a beam current of 1 nA. The samples were drop cast onto a Cu 200 carbon/formvar coated grid (Ted Pella, Redding, Ca, USA) and negatively stained using a 2% uranyl actetate solution for 2 min. Images were captured using a Ceta-S camera and analysed using Velox Analytical Software (Thermo Fisher Scientific Electron Microscopy Solutions, Hillsboro, USA, (TFS)).

Chemical Synthesis

Clickable alkyne-functionalized Y-shaped compound 3

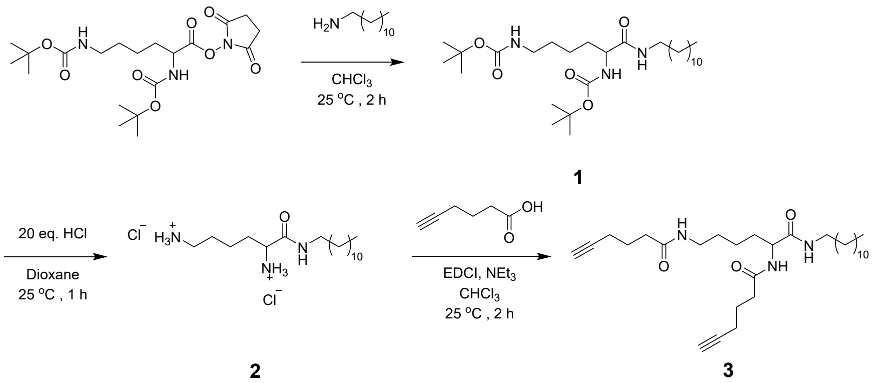

Scheme S1. Synthesis of alkyne-functionalized Y-shaped compound **3**.

The synthesis of clickable alkyne-functionalized Y-shaped compound **3** followed the exact same protocol as we described previously.[3] Firstly, to a flask containing $N^{\alpha,\varepsilon}$-bis-Boc-*L*-lysine *N*-hydroxysuccinimide ester (6.32 g, 14.2 mmol) dissolved in chloroform (120 mL), was added dodecylamine (2.90 g, 15.6 mmol). The reaction mixture was stirred at 25 °C for 20 h, and subsequently washed against water (200 mL). The organic phase was dried over $MgSO_4$ and basic alumina, filtered, and concentrated in vacuo. The concentrated solution was then percolated over a column of silica gel plug using ethyl acetate as the eluent. The collected fraction was dried in vacuo to yield compound **1** as a white solid (7.80 g, 87 mol% yield). ^{1}H NMR (400 MHz, $CDCl_3$, 25 °C): δ_H (ppm) = 6.39 (br s, 1H, C(=O)-N*H*), 5.24 (br s, 1H, C(=O)-N*H*-CH), 4.67-4.70 (t, 1H, C(=O)-NH-CH_2), 4.02-4.03 (d, 1H, C*H*-C(=O)), 3.19-3.24 (m, 2H, CH-C(=O)-NH-CH_2), 3.09-3.11 (d, 2H, O-C(=O)-NH-CH_2), 1.59-1.82 (m, 2H, CH_2-CH), 0.90-0.87 (m, 42H, aliphatic CH_2, O-C-(CH_3)$_3$, CH_2-CH$_2$-CH$_2$-CH, CH$_2$-CH_2-CH$_2$-CH), 0.90-0.87 (t, 3H, CH$_2$-CH_3). ^{13}C NMR (400 MHz, $CDCl_3$, 25 °C): δ_C (ppm) = 171.98, 156.17, 155.84, 79.91, 79.08, 54.43, 39.49, 31.89, 29.63, 29.60, 29.58, 29.52, 29.32, 29.28, 28.43, 28.32, 26.87, 22.66, 14.08.

The Boc groups of compound **1** were then deprotected under acidic conditions. Specifically, compound **1** (5.20 g, 10.1 mmol (20.4 mmol of Boc groups)) was dissolved in dioxane (20 mL), followed by the addition of 4 M HCl solution in dioxane (ca. 100 mL, 400 mmol of HCl). The contents were stirred at 25 °C for 1 h in which white precipitates formed. The contents were concentrated in vacuo to which hexane (ca. 300 mL) was added to induce further precipitation. The solids were recovered by centrifugation and washed once more with hexane before drying in vacuo for several days at 40 °C to give the hygroscopic compound **2** as an off-yellow solid (3.40 g, 86 mol% yield). ^{1}H NMR (400 MHz, DMSO-d_6, 25 °C): δ_H (ppm) = 8.67-8.69 (t, 1H, C(=O)-NH), 8.14-8.33 (d, 6H, (NH_3^+)$_6$), 3.74 (s, 1H, CH-C(=O)), 3.08-3.11 (t, 2H, NH-CH_2), 2.73-2.74 (d, 2H, NH_3^+-CH_2), 1.71-1.76 (q, 2H, NH_3^+-CH_2-CH_2), 1.58-1.61 (t, 2H, CH_2-CH), 1.34-1.44 (m, 20H, aliphatic CH_2), 0.84-0.87 (t, 3H, CH_2-CH_3). ^{13}C NMR (400 MHz, DMSO-d_6, 25 °C): δ_C (ppm) = 168.63, 52.32, 38.62, 31.77, 30.77, 29.53, 29.50, 29.26, 29.19, 26.85, 26.63, 22.56, 22.61, 14.43.

5-hexynoic acid (861 µL, 8.5 mmol) and EDCI (1.63 g, 8.5 mmol) were added into a round bottom flask and subsequently dissolved with chloroform (30 mL). Compound **2** (1.49 g, 7.7 mmol of amine) was added to the flask followed by triethylamine (1.35 mL, 9.7 mmol). The contents were stirred at 25 °C for 2 h. Next, the contents were washed against 0.01 M HCl solution (1 × 30 mL), saturated $NaHCO_3$ (1 × 30 mL), and brine (1 × 30 mL). The organic phase was mixed with $MgSO_4$, filtered, and concentrated in vacuo. The crude product was further purified by column chromatography on silica gel using ethyl acetate as the eluent (R_f ~ 0.35) to yield the alkyne-functionalized Y-shaped compound **3** as an off-yellow solid (0.70 g, 36 mol% yield). ^{1}H NMR (400 MHz, DMSO-d_6, 25 °C): δ_H (ppm) = 7.76-7.89 (m, 3H, C(=O)-NH), 4.13-4.18 (q, 1H, CH-C(=O)), 2.97-3.06 (m, 4H, C(=O)-NH-CH_2), 2.73-2.74 (t, 2H, CH≡C-CH_2), 2.12-2.23 (m, 8H, CH≡C-CH_2, CH_2-C(=O)), 1.55-1.69 (m, 4H, -CH_2-CH_2-C(=O)), 1.46-1.49 (m, 2H, CH_2-CH), 1.37 (s, 4H, CH_2-CH_2-CH_2-CH, CH_2-CH_2-CH_2-CH),

1.24 (s, 20H, aliphatic CH_2), 0.84-0.87 (t, 3H, CH_2-CH_3). ^{13}C NMR (400 MHz, DMSO-d_6, 25 °C): δ_C (ppm) = 171.98, 171.88, 171.66, 84.51, 84.48, 71.78, 71.75, 52.91, 38.85, 38.75, 34.65, 34.48, 32.29, 31.79, 29.56, 29.51, 29.32, 29.22, 29.21, 26.79, 24.80, 24.78, 23.32, 22.58, 17.90, 17.86, 14.39.

Azide-functionalized compound 6

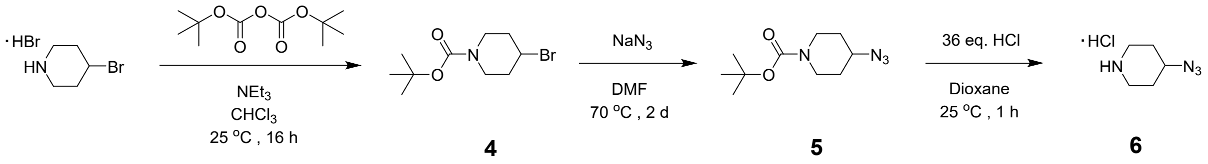

Scheme S2. Synthesis of azide-functionalized compound **6**.

The synthesis of precursors **4** and **5** followed the exact same protocol as previously described. [4] Firstly, a suspension of 4-bromopiperidine hydrobromide (2.45 g, 10.0 mmol) in chloroform (70 mL) was prepared, followed by the addition of di-*tert*-butyl dicarbonate (2.60 g, 12.0 mmol). After the carbonate was fully solubilized, triethylamine (1.46 mL, 10.5 mmol) was added into the reaction mixture. The suspended salt gradually dissolved upon stirring and a clear reaction mixture was obtained. The contents were stirred for 16 h at 25 °C. Next, the contents were washed against water (2 × 50 mL). The organic phase was then mixed with $MgSO_4$ and basic alumina, filtered, and dried in vacuo to yield compound **4** as a light-yellow oil (2.22 g, 84 mol% yield). It is noteworthy that the product still contained some of the reactant di-*tert*-butyl dicarbonate (ca. 10 mol% compared to **4** as determined via ^{1}H NMR analysis), though this did not affect the subsequent reaction step. ^{1}H NMR (400 MHz, $CDCl_3$, 25 °C): δ_H (ppm) = 4.34 (m, 1H, CH-Br), 3.30-3.72 (m, 4H, N-(CH_2)$_2$), 1.92-2.13 (m, 4H, (CH_2)$_2$-CH), 1.48 (s, 9H, (CH_3)$_3$-C). ^{13}C NMR (400 MHz, $CDCl_3$, 25 °C): δ_C (ppm) = 154.65, 85.18, 79.82, 49.53, 35.60, 28.41.

An azidation step was performed to convert the halogen into clickable azide group. Compound **4** (2.20 g, 8.33 mmol) was dissolved in DMF (30 mL), followed by the addition of sodium azide (4.33 g, 66.7 mmol). The contents were stirred at 70 °C for 2 days. After the reaction, ethyl acetate (100 mL) and diethyl ether (40 mL) were added, and the organic phase was washed with water (3 × 120 mL). The organic phase was then mixed with Na_2SO_4, filtered, and concentrated in vacuo. The crude product was further purified by column chromatography on silica gel using hexane:ethyl acetate 9:1 as the eluent ($R_f \sim 0.38$) to isolate the elimination by-product to yield compound **5** as a pale yellow liquid (1.34 g, 71 mol% yield). ^{1}H NMR (400 MHz, $CDCl_3$, 25 °C): δ_H (ppm) = 3.80-3.85 (m, 2H, N-CH_2), 3.56-3.60 (m, 1H, CH-N_3), 3.06-3.13 (m, 2H, N-CH_2), 1.52-1.89 (m, 4H, $(CH_2)_2$-CH), 1.47 (s, 9H, $(CH_3)_3$-C). ^{13}C NMR (400 MHz, $CDCl_3$, 25 °C): δ_C (ppm) = 154.62, 79.80, 57.57, 41.36, 30.58, 28.40.

The azide-functionalized compound **6** was synthesized by removing the Boc groups of compound **5**. Specifically, **5** (250 mg, 1.11 mmol) was dissolved in dioxane (2 mL), followed by the addition of 4 M HCl solution in dioxane (10 mL, 40 mmol). After 1 h, diethyl ether (90 mL) was added to precipitate the product. The precipitates were isolated from the supernatant via centrifugation and redissolved in methanol (2 mL). The solution was then precipitated once more in diethyl ether (20 mL), followed by drying in vacuo to yield the azide compound **6** as a white solid (130 mg, 72 mol% yield). ^{1}H NMR (400 MHz, $CDCl_3$, 25 °C): δ_H (ppm) = 9.66 (br s, 2H, NH_2^+), 3.86-3.93 (m, 1H, CH-N_3), 3.17-3.37 (m, 4H, NH_2^+-$(CH_2)_2$), 1.98-2.32 (m, 4H, $(CH_2)_2$-CH-N_3). ^{13}C NMR (400 MHz, $CDCl_3$, 25 °C): δ_C (ppm) = 54.06, 40.35, 27.05.

β-Lactamase-responsive BLM-Dendron

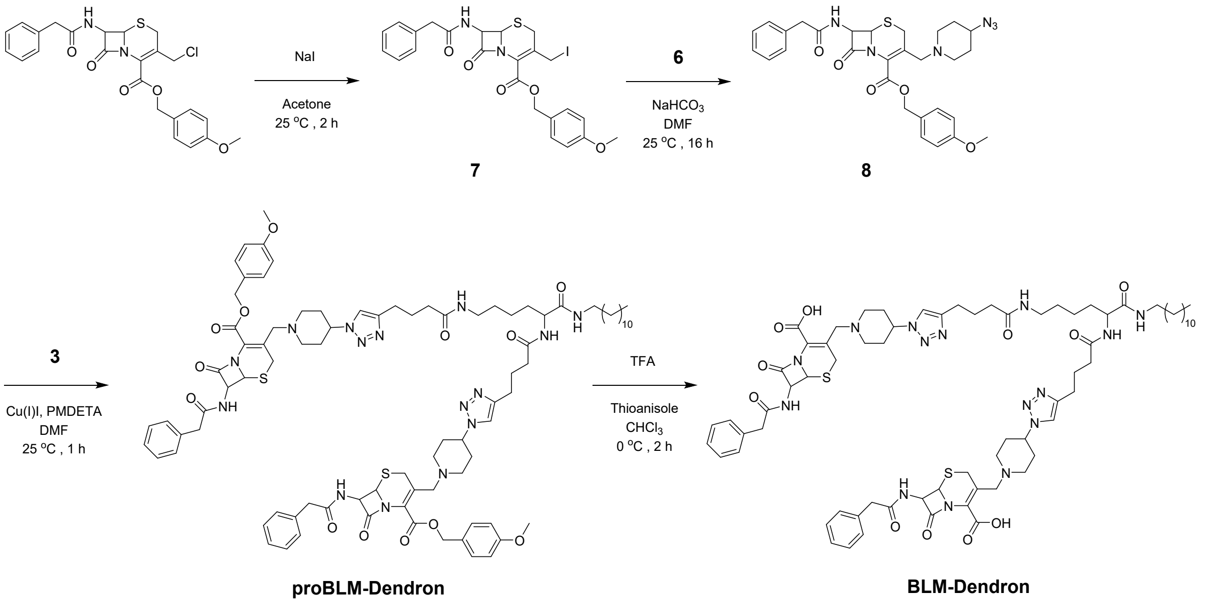

Scheme S3. Synthesis of β-Lactamase-responsive amine-caged antimicrobial compound **BLM-Dendron**.

3-Chloromethyl-7-phenylacetylamino cephalosporanic acid *p*-methoxybenzyl ester (1 g, 2.05 mmol) and NaI (379.5 mg, 2.53 mmol) were dissolved with acetone (20 mL) in a round bottom flask, and the reaction mixture was subsequently stirred at 25 °C for 2 h.[5] Dichloromethane (20 mL) was then added to the solution and the organic layer was washed against 5% $Na_2S_2O_3$ solution (8 mL), dehydrated over $MgSO_4$, filtered, and dried in vacuo to yield the iodized compound **7** as a yellow solid (1.10 g, 92 mol% yield). ^{1}H NMR (400 MHz, $CDCl_3$, 25 °C): δ_H (ppm) = 7.27-7.42 (m, 7H, C_6H_5-CH_2-C(=O), C-$(CH)_2$-$(CH)_2$-C-O), 6.90-6.92 (d, 2H, C-$(CH)_2$-$(CH)_2$-C-O), 6.10-6.12 (d, 1H, C(=O)-N*H*), 5.77-5.81 (q, 1H, NH-C*H*), 5.24 (s, 2H, O-CH_2-C_6H_4), 4.92-4.93 (d, 1H, C*H*-S-CH_2), 4.31-4.39 (q, 2H, CH_2-I), 3.83 (s, 3H, O-CH_3), 3.71-3.75 (d, 1H, CH-S-CH_2), 3.65-3.67 (d, 2H, C_6H_5-CH_2-C(=O)), 3.43-3.48 (d, 1H, CH-S-CH_2). ^{13}C NMR (400 MHz, $CDCl_3$, 25 °C): δ_C (ppm) = 171.15, 164.06, 161.39, 159.97, 133.56,

130.78, 129.46, 129.26, 128.99, 127.83, 126.71, 123.27, 114.01, 68.06, 59.07, 57.92, 55.29, 43.35, 28.41, 3.66.

Next, compounds **7** (514 mg, 0.89 mmol), **6** (120 mg, 0.74 mmol) and $NaHCO_3$ (155 mg, 1.85 mmol) were dissolved in DMF (15 mL), and the solution was stirred at 25 °C for 16 h. Chloroform (20 mL) was then added into the solution, followed by washing against saturated brine, dried over $MgSO_4$, filtered, and concentrated. The crude product was further purified by column chromatography on silica gel using hexane:ethyl acetate (7:5) solvent mixture as the eluent ($R_f \sim 0.25$) to yield the azide-functionalized compound **8** as a yellow solid (154 mg, 36 mol% yield). ^{1}H NMR (400 MHz, $CDCl_3$, 25 °C): δ_H (ppm) = 7.28-7.41 (m, 7H, C_6H_5-CH_2-C(=O), C-(CH)$_2$-(CH)$_2$-C-O), 6.90-6.92 (m, 2H, C-(CH)$_2$-(CH)$_2$-C-O), 6.07-6.09 (d, 1H, C(=O)-NH), 5.80-5.82 (q, 1H, NH-CH), 5.20 (s, 2H, O-CH_2-C_6H_4), 4.93-4.95 (d, 1H, CH-S-CH_2), 3.83 (s, 3H, O-CH_3), 3.65-3.68 (d, 2H, C_6H_5-CH_2-C(=O)), 3.60-3.61 (t, 1H, CH-N_3), 3.13-3.41 (m, 4H, CH-S-CH_2, CH_2-N-(CH_2)$_2$), 2.13-2.65 (d, 4H, CH_2-N-(CH_2)$_2$), 1.67-1.84 (d, 4H, (CH_2)$_2$-CH-N_3). ^{13}C NMR (400 MHz, $CDCl_3$, 25 °C): δ_C (ppm) = 171.12, 164.66, 161.86, 159.98, 133.62, 130.78, 130.23, 129.54, 129.50, 129.24, 129.14, 127.78, 113.99, 67.85, 59.17, 57.83, 55.31, 53.78, 50.84, 43.38, 30.86, 26.95.

Thereafter, compound **8** was clicked with **3** via click chemistry to produce the *p*-methoxy benzyl-protected compound **proBLM-Dendron**. Compounds **8** (131.4 mg, 0.23 mmol) and **3** (55 mg, 0.115mmol) were dissolved in DMF (3 mL) in a round bottom flask. Copper (I) iodide (43.8 mg, 0.23 mmol) was then added into the flask and the mixture was degassed with N_2 for 10 min. Then, PMDETA (48 μL, 0.23 mmol) was added into the flask under N_2 atmosphere and the system was degassed with N_2 for another 10 min. The mixture was stirred at room temperature for 1 h under N_2 atmosphere. Afterwards, chloroform (25 mL) was mixed into the flask, and the combined organic phase was washed against 0.02 M EDTA solution (2 × 10 mL), followed by water (3 × 20 mL). The organic phase was subsequently dried over $MgSO_4$,

filtered, and concentrated in vacuo. The concentrated solution was precipitated into diethyl ether (40 mL). After removing the supernatant by centrifugation, the product was dried in vacuo to yield compound **9** as a brown solid (138 mg, 73 mol% yield). ^{1}H NMR (400 MHz, DMSO-d_6, 25 °C): δ_H (ppm) = 9.12-9.15 (d, 2H, C_6H_5-CH_2-C(=O)-N*H*), 7.75-7.96 (m, 5H, C*H*-N_3, CH_2-C(=O)-N*H*-CH_2, CH_2-C(=O)-N*H*-CH, CH-C(=O)-N*H*), 7.21-7.37 (m, 14H, C_6H_5-CH_2, C-(C*H*)$_2$-(CH)$_2$-C-O-CH_3), 6.93-6.96 (d, 4H, C-(CH)$_2$-(C*H*)$_2$-C-O-CH_3), 5.66-5.69 (m, 2H, C_6H_5-CH_2-C(=O)-NH-C*H*), 5.12-5.25 (m, 6H, C*H*-S-CH_2, CH_2-C_6H_4-O-CH_3), 4.32-4.37 (m, 2H, (CH_2)$_2$-C*H*-N_3), 4.15-4.21 (q, 1H, CH_2-C*H*-C(=O)-NH), 3.75 (s, 6H, C_6H_4-O-CH_3), 3.47-3.61 (m, 8H, C_6H_5-CH_2, CH-S-CH_2), 3.13-3.22 (q, 4H, C-CH_2-N-(CH_2)$_2$), 2.98-3.06 (m, 4H, CH_2-C(=O)-NH-CH_2, CH-C(=O)-NH-CH_2), 2.80 (s, 4H, CH_2-N-(CH_2)$_2$), 2.56-2.59 (t, 4H, N_3-CH_2-CH_2), 1.78-2.20 (m, 20H, CH_2-N-(CH_2)$_2$, (CH_2)$_2$-CH-N_3, N_3- CH_2-CH_2-CH_2, N_3-CH_2-CH_2-CH_2), 1.63-1.66 (m, 2H, CH_2-CH-C(=O)-NH), 1.36 (s, 4H, CH_2-CH_2-CH_2-CH-C(=O)-NH, CH_2-CH_2-CH_2-CH-C(=O)-NH), 1.22 (s, 20H, aliphatic CH_2), 0.84-0.86 (t, 3H, (CH_2)$_{10}$-CH_3). ^{13}C NMR (400 MHz, DMSO-d_6, 25 °C): δ_C (ppm) = 172.17, 171.99, 171.39, 165.25, 162.44, 159.85, 136.28, 130.93, 129.46, 128.69, 127.60, 127.45, 126.95, 124.84, 120.45, 114.29, 79.65, 67.35, 59.52, 58.41, 58.04, 57.52, 55.60, 52.29, 51.67, 42.06, 38.85, 35.40, 35.07, 32.36, 31.76, 29.52, 29.48, 29.18, 26.77, 25.71, 25.24, 25.11, 22.56, 14.43.

The removal of the *p*-methoxy benzyl group on **proBLM-Dendron** proceeded by firstly dissolving **proBLM-Dendron** (116 mg, 0.070 mmol) in chloroform (3 mL), followed by the addition of thioanisole (328 μL) and TFA (1.2 mL). The solution was stirred at 0 °C for 2 h. Afterwards, diethyl ether (40 mL) was added to the solution to precipitate the products. The precipitates were isolated from the supernatant after centrifugation and redissolved in chloroform (4 mL). The solution was then precipitated once more in diethyl ether (40 mL), and the precipitates were dried in vacuo to yield the final product **BLM-Dendron** as a yellow solid (83 mg, 84 mol% yield). ^{1}H NMR (400 MHz, DMSO-d_6, 25 °C): δ_H (ppm) = 9.11-9.13 (d, 2H,

C_6H_5-CH_2-C(=O)-N*H*), 7.75-7.98 (m, 5H, C*H*-N_3, CH_2-C(=O)-N*H*-CH_2, CH_2-C(=O)-N*H*-CH, CH-C(=O)-N*H*), 7.20-7.32 (m, 10H, C_6H_5-CH_2), 5.62-5.66 (m, 2H, C_6H_5-CH_2-C(=O)-NH-C*H*), 5.07-5.08 (d, 2H, C*H*-S-CH_2), 4.55-4.61 (q, 2H, $(CH_2)_2$-C*H*-N_3), 4.15-4.20 (q, 1H, CH_2-C*H*-C(=O)-NH), 3.47-3.62 (m, 8H, C_6H_5-CH_2, CH-S-CH_2), 3.35 (s, 2H, C(=O)-O*H*), 2.98-3.12 (m, 8H, C-CH_2-N-$(CH_2)_2$, CH_2-C(=O)-NH-CH_2, CH-C(=O)-NH-CH_2), 2.57-2.59 (t, 12H, CH_2-N-(CH_2)$_2$, N_3-CH_2-CH_2), 2.03-2.18 (m, 12H, (CH_2)$_2$-CH-N_3, CH_2-C(=O)-NH-CH_2-CH_2, CH_2-C(=O)-NH-CH_2), 1.76-1.84 (m, 4H, N_3-CH_2-CH_2), 1.43-1.64 (m, 2H, CH_2-CH-C(=O)-NH), 1.23 (s, 20H, aliphatic CH_2), 0.83-0.87 (q, 3H, $(CH_2)_{10}$-CH_3). ^{13}C NMR (400 MHz, DMSO-d_6, 25 °C): δ_C (ppm) = 172.17, 172.04, 171.44, 165.02, 136.28, 129.47, 128.70, 126.97, 120.89, 59.60, 58.20, 52.90, 42.04, 38.85, 35.37, 31.76, 29.52, 29.48, 29.18, 26.77, 25.67, 25.19, 25.08, 23.34, 22.56, 14.43.

***Secondary cyclic amine* Ref-Dendron**

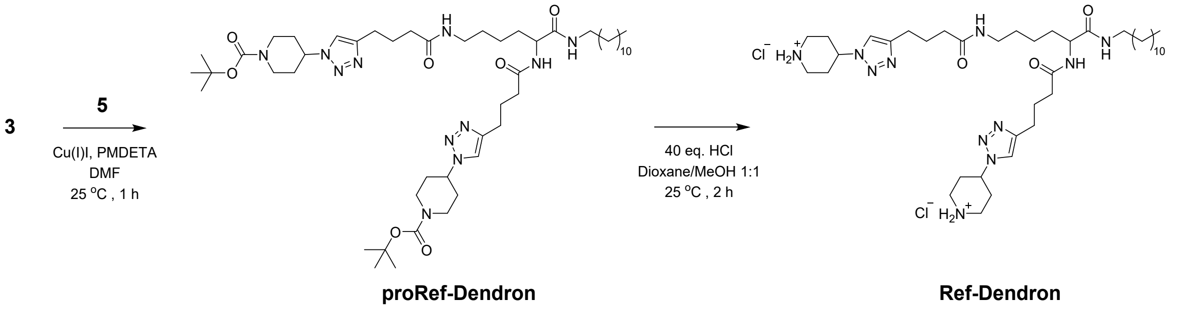

Scheme S4. Synthesis of **Ref-Dendron**.

The synthesis of **Ref-Dendron** proceeded in the same manner as previously described.[4] For the click reaction, the azide **5** (1 mol eq. and at 1 mmol basis) and the alkyne **3** (1 mol eq.) were solubilized in dimethylformamide (2 mL per 1 mmol of azide). Copper (I) iodide (1 mol eq.) was then added into the flask and the contents were degassed with N_2 for 10 min. Next, PMDETA (1 mol eq.) was added into the flask under N_2 atmosphere, and the contents were

degassed with N_2 for another 10 min. The reaction mixture was stirred at 25 °C for 1 h under N_2 atmosphere. Afterwards, chloroform (ca. 25 mL) was added into the flask and the combined organic phase was washed against 0.02 M EDTA solution (2 × 20 mL) and water (1 × 20 mL). The organic phase was subsequently mixed with $MgSO_4$ and basic alumina, filtered, and dried in vacuo to yield a wet solid that contained trace amounts of DMF. The high boiling point solvent was removed from the wet solid by mixing with diethyl ether (25 mL), followed by centrifugation and decanting steps to discard the filtrate. The recovered solids were then dried in vacuo to yield **proRef-Dendron** as a pale-yellow solid (typically 50 mol% yield). ^{1}H NMR (400 MHz, DMSO-d_6, 25 °C): δ_H (ppm) = 7.95 (d, 2H, CH-C(=O)-N*H*, C(=O)-N*H*- CH), 7.84-7.88 (t, 2H, C$H_2$$N_3$), 7.77 (s, 1H, C(=O)-N*H*), 4.61-4.67 (m, 1H, $(CH_2)_2$-C*H*), 4.16-4.18 (d, 1H, C*H*-C(=O)-NH), 4.02-4.05 (d, 4H, N-(CH_2)$_2$), 2.99-3.00 (d, 4H, C(=O)-NH-CH_2, CHC(=O)-NH-CH_2), 2.55-2.59 (t, 4H, N_3CH_2), 2.15-2.19 (t, 4H, CH_2CHC(=O)), 2.02-2.09 (m, 4H, (CH_2)$_2$-CH), 1.79-1.81 (d, 6H, C$H_2$$CH_2$C(=O), C$H_2$CH-C(=O)-NH), 1.57 (s, 2H, C$H_2$$CH_2CH_2$CHC(=O)), 1.42 (s, 18H, (CH_3)$_3$-C), 1.34 (s, 2H, CH_2C$H_2$$CH_2$CHC(=O)), 1.22 (s, 20H, aliphatic CH_2), 0.83-0.86 (t, 3H, CH_2CH_3). ^{13}C NMR (400 MHz, DMSO-d_6, 25 °C): δ_C (ppm) = 172.20, 172.03, 154.26, 146.78, 146.77, 120.53, 120.50, 79.39, 57.28, 52.91, 38.86, 38.74, 35.38, 35.04, 32.36, 31.75, 29.50, 29.46, 29.34, 29.16, 28.52, 26.75, 25.64, 25.59, 25.21, 25.07, 23.34, 22.54, 14.39.

Meanwhile for the Boc deprotection step, the Boc-protected **proRef-Dendron** (1 mol eq. and at 0.4 mmol basis) were dissolved in a 1,4-dioxane:methanol 1:1 solvent mixture (1 mL per 50 mg of starting material), followed by the addition of 4 M HCl solution in 1,4-dioxane (40 mol eq.). The contents were stirred at 25 °C for 2 h, during which precipitates were formed. Diethyl ether (20 mL) was added to the mixture at the end of the reaction. The precipitates were isolated from the supernatant after centrifugation and dried in vacuo to yield **Ref-Dendron** as a pale-yellow solid (80 mol% yield). ^{1}H NMR (400 MHz, DMSO-d_6, 25 °C): δ_H

(ppm) = 8.86-9.04 (t, 4H, (NH_2^+)$_2$), 7.88-7.94 (q, 5H, C$H_2$$N_3$, C(=O)-N*H*), 4.73-4.79 (m, 2H, (CH_2)$_2$-C*H*), 4.12-4.13 (d, 1H, C*H*-C(=O)-NH), 3.36-3.40 (m, 4H, C(=O)-NH-CH_2), 2.97-3.10 (m, 8H, (NH_2^+)-(CH_2)$_2$), 2.55-2.59 (m, 4H, N_3CH_2), 2.08-2.28 (m, 12H, (CH_2)$_2$-CH, CH_2C(=O)), 1.77-1.80 (t, 4H, C$H_2$$CH_2$C(=O)), 1.46-1.57 (m, 2H, CH_2CHC(=O)), 1.34-1.35 (d, 4H, C$H_2$$CH_2$$CH_2$CHC(=O), CH_2C$H_2$$CH_2$CHC(=O)), 1.19 (s, 20H, aliphatic CH_2), 0.80-0.84 (t, 3H, CH_2CH_3). ^{13}C NMR (400 MHz, DMSO-d_6, 25 ^{o}C): δ_C (ppm) = 172.60, 172.53, 172.26, 146.85, 121.19, 121.16, 54.56, 53.13, 42.49, 38.90, 38.76, 35.33, 35.01, 32.11, 31.68, 29.42, 29.38, 29.35, 29.14, 29.08, 28.96, 26.66, 25.59. 25.52, 24.97, 24.85, 23.26, 22.49, 14.36.

Biological Experiments

Minimum inhibitory concentration (MIC) determination

The MICs of the compounds were evaluated by the broth microdilution method against several pathogens including *Pseudomonas aeruginosa* ATCC 27853, *Escherichia coli* K12 and *Staphylococcus aureus* ATCC 29213 as well as three MDR *P. aeruginosa* strains (PA 31, PA34 and PA 37) according to the Clinical and Laboratory Standards Institute (CLSI) guidelines. This was to evaluate the bacteriostatic efficacy of the compounds. Briefly, a single bacterial colony was cultured in 10 mL of Mueller–Hinton broth (MHB) at 37 °C with 200 rpm shaking overnight. Subsequently, a subculture was prepared from the overnight culture by diluting 1:100 in 10 mL MHB and allowed to grow to mid-log phase, then diluted to the appropriate concentration for the MIC test. A twofold dilution series of 100 µL of compounds solution in PBS were added into 96-well microplates (Costar, Corning), followed by the addition of 50 µL of the β-lactamase enzyme, and 50 µL of the subculture suspension. The final concentration of bacteria in each well was ca. 5×10^5 cells mL^{-1}. After incubating the plate at 37 °C for 20 h, the absorbance at 600 nm was measured using a microtiter plate reader (FLUOstar Omega, BMG Labtech). MIC values were defined as the lowest concentration of samples that showed no visible bacteria growth and inhibited more than 90% bacteria growth. Positive controls without compounds and negative controls without bacteria were included. All assays included duplicates and were repeated in at least three independent experiments.

Planktonic bacteria killing study

A time-kill study was carried out to evaluate the bactericidal efficiency of the compounds where *P. aeruginosa* ATCC 27853 was used as the target strain and the bacterial suspensions were prepared in the same manner as with the MIC assay. Two different concentrations were used for each compound (1 × MIC and 2 × MIC), and the compounds were incubated with

bacterial suspension for predetermined times (1, 4 and 20 h). The viability of planktonic cells was then determined by a drop plate method where the planktonic cells were serially diluted in sterile PBS and plated onto Luria Bertani (LB) agar. After 24 h of incubation at 37 °C, bacteria colonies were counted and colony forming unit (CFU) analysis was performed. All assays included two replicates and were repeated in at least three independent experiments.

Biofilm bacteria killing study

The biofilm bacteria killing study was conducted against *P. aeruginosa* ATCC 27853. The bacterial suspension (ca. 1×10^7 cells mL^{-1}) was prepared by diluting the overnight culture (as prepared in the same manner above) 1:200 in M9 minimal medium (48 mM Na_2HPO_4, 22 mM KH_2PO_4, 9 mM NaCl, and 19 mM NH_4Cl, pH 7.0, freshly supplemented with 2 mM $MgSO_4$, 100 µM $CaCl_2$, and 20 mM glucose). The bacterial suspension (1 mL) was then aliquoted into each well of the tissue-culture treated 24-well plates (Costar, Corning). The biofilm was preformed by incubating the plates for 6 h at 37 °C and 180 rpm. The supernatant of each well was pipetted into separate tubes to which the compounds were added at different concentrations (1, 2, and 4 × MIC). The solutions were then vortexed and subsequently transferred back to the wells containing the preformed biofilms, followed by 1 h incubation at 37 °C and 180 rpm. After treatment, the biofilm cell viability was determined by a drop plate method. Cells attached on the interior surfaces of the well (surface area = 4.5 cm^2) were washed twice with PBS to remove loosely attached bacteria, before being resuspended and homogenized in PBS by incubation in an ultrasonication bath (150 W, 40kHz; Unisonics) for 20 min. Resuspended biofilm cells were then serially diluted and plated onto LB agars. After 24 h of incubation at 37 °C, biofilm colonies were counted and CFU analysis was performed. All assays included two replicates and were repeated in at least three independent experiments.

Outer membrane permeability study

The influence of the compounds on outer membrane permeability was examined based on a previous study.[6] Briefly, a concentrated bacteria suspension (*P. aeruginosa* ATCC 27853, ca. 1×10^9 cells mL^{-1}) was prepared and resuspended in PBS as described above. After 10 min of incubation at 37 °C and 180 rpm with the compounds at their respective 1 × MIC concentration, the cells were collected via centrifugation (3800 rpm, 10 min) and resuspended in PBS containing 0.5% sodium deoxycholate. The suspension was further incubated for 10 min at 37 °C and 180 rpm. After vortex, 200 µL of the suspension was transferred to 96-well plates and the extent of cell lysis (optical density at 485 nm) was measured using a plate reader (FLUOstar Omega, BMG Labtech). Negative control sample (i.e., PBS only) and background were used as reference points to determine the relative difference in optical density of other samples via the following equation:

$$\%\ Optical\ density = \frac{Abs_{485\ nm}^{Sample} - Abs_{485\ nm}^{Background}}{Abs_{485\ nm}^{Negative\ control} - Abs_{485\ nm}^{Background}} \times 100\%$$

All assays included duplicates and were repeated in at least two independent experiments.

Inner membrane permeability study

A membrane disruption kinetic assay with temporal resolution was applied based on the propidium iodide (PI) staining method. To initiate the assay, a concentrated bacteria suspension of *P. aeruginosa* ATCC 27853 in PBS (ca. 4×10^8 cells mL^{-1}) was prepared via centrifugation (3800 rpm, 10 min) and subsequent resuspension. The compounds at five different concentrations (0.25 × MIC, 0.5 × MIC, 1 × MIC, 2 × MIC, and 4 × MIC) were then loaded on a flatbottom, black cell-culture-grade 96-well plate (Costar, Corning), followed by the addition of PI (5 µmol, Sigma-Aldrich) and bacteria suspension. The fluorescence intensity was monitored kinetically for 1 h using a microtiter plate reader (FLUOstar Omega, BMG Labtech) with excitation and emission wavelengths of 544 nm and 620 nm, respectively. Positive

controls with **Ref-Dendron** and negative controls without antimicrobial were included. The fluorescence intensity was normalized by the ratio to negative control. Datasets were presented as heat maps with temporal and concentration resolutions using MATLAB. All assays included duplicates and were repeated in at least two independent experiments.

Hemolysis study

Hemolytic activity of the compounds was determined on fresh defibrinated sheep blood (Serum Australis). The sheep blood was diluted 1:20 in PBS (pH 7.4), pelleted by centrifugation, and washed three times in PBS (3800 *rpm*, 9 min). The red blood cells (RBCs) were then collected and resuspended to achieve 5% (v/v) in PBS. A twofold dilution series of 250 μL of compounds solution in PBS were prepared in sterilized tubes, followed by the addition of RBC suspensions (250 μL). PBS and Triton-X 100 (1% v/v in PBS) were used as the negative and positive controls, respectively. After 2 h incubation at 37 °C and 180 rpm, the tubes were centrifuged (1500 rpm, 9 min), and aliquots of the supernatants (100 μL) were transferred into a 96-well microplate, where the absorbance values were monitored at 485 nm using a microtiter plate reader (FLUOstar Omega, BMG Labtech). The percentage of haemolysis was calculated based on the absorbance values and the formula below:

$$\%\ \text{Hemolysis} = (\text{Abs}_{\text{compound}} - \text{Abs}_{\text{negative}}) / (\text{Abs}_{\text{positive}} - \text{Abs}_{\text{negative}}) \times 100\%$$

All assays include duplicates and were repeated in at least three independent experiments.

Mammalian cell viability assay

The mammalian cell viability study was determined by PrestoBlue (Thermo Fisher Scientific) assay on murine embryonic fibroblasts (MEF) CF-1 (ATCC SCRC-1040), which was kindly provided by the Cell Culture Facility of the Mark Wainwright Analytical Centre at UNSW. Using a cell culture incubator (Eppendorf CellXpert C170i), MEF cells were cultured to

subconfluency at 37 °C and 5% CO_2 in Dulbecco's Modified Eagle's Medium (DMEM, Gibco) that was supplemented by L-glutamine (2 mmol, Sigma-Aldrich) and fetal bovine serum (10% (v/v), Sigma-Aldrich). For the cell viability assay, MEF cells were subcultured twice and diluted to a final concentration of 5×10^4 cells mL^{-1} in DMEM. After adding 100 μL of cell suspension to each well of a flat-bottom, black cell-culture-grade 96-well plate (Costar, Corning), the plate was cultured for 20 h at 37 °C and 5% CO_2 to attach the cells. Then, the supernatant was aspirated, and 50 μL of fresh DMEM was added, followed by another 50 μL of the compounds in DMEM. Subsequently, the plate was incubated for 24 h at 37 °C and 5% CO_2. The fluorescence was measured (excitation 560 nm and emission 590 nm) using a microplate reader (CLARIOstar, BMG Labtech) after adding 10 μL of PrestoBlue agent in each well and culturing for 2 h at 37 °C and 5% CO_2. The percentage of cell viability was determined based on the fluorescence of the samples relative to that of the cells only control sample. All assays include duplicates and were repeated in at least three independent experiments.

SUPPORTING RESULTS

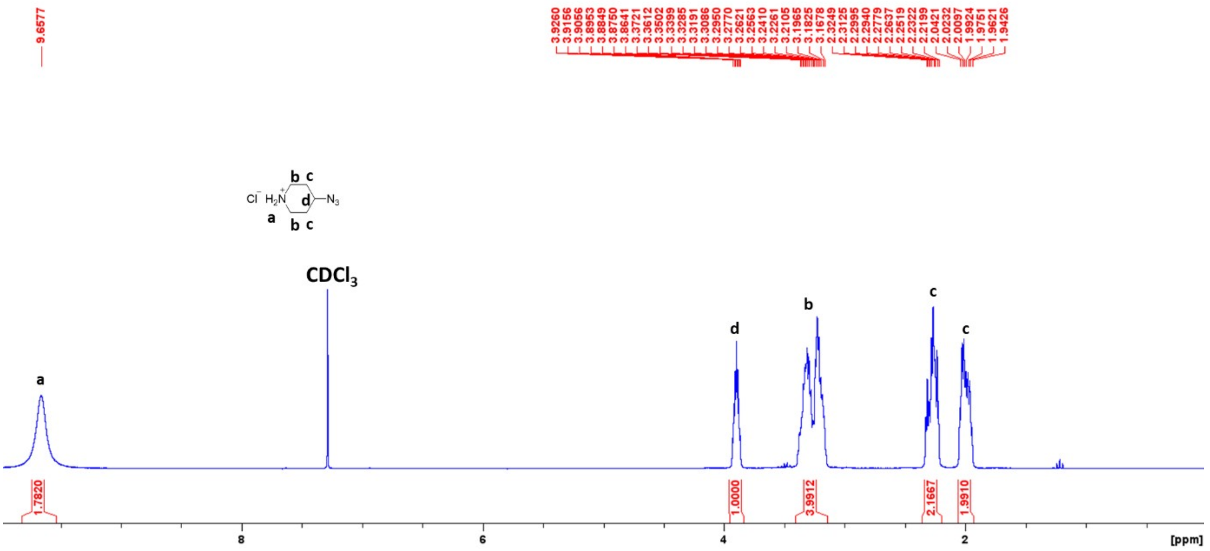

Figure S1. ^{1}H NMR spectra of compound **6** in $CDCl_3$.

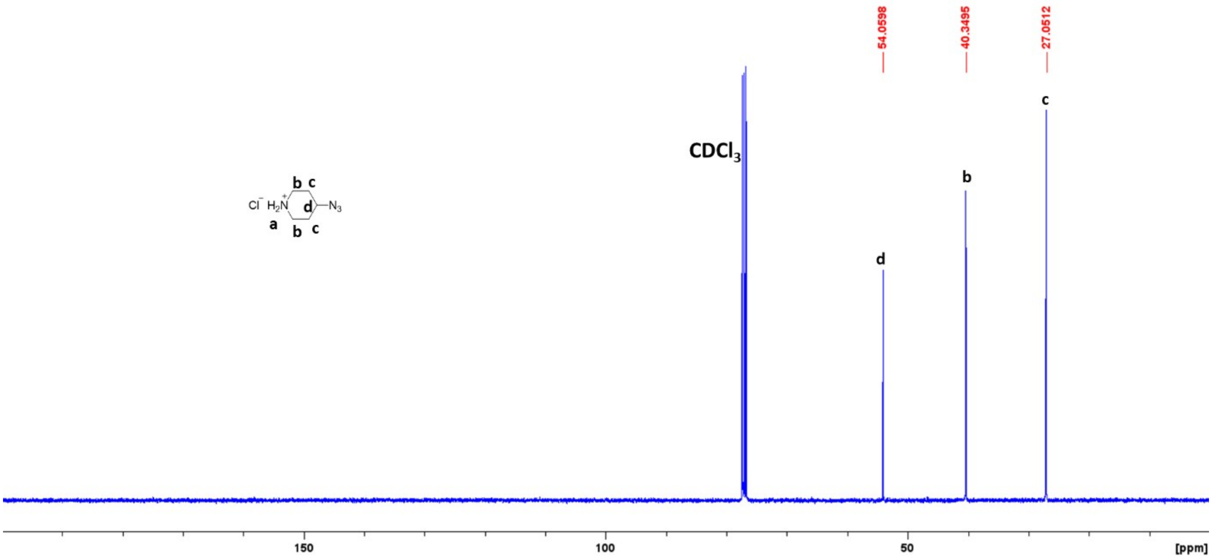

Figure S2. ^{13}C NMR spectra of compound **6** in $CDCl_3$.

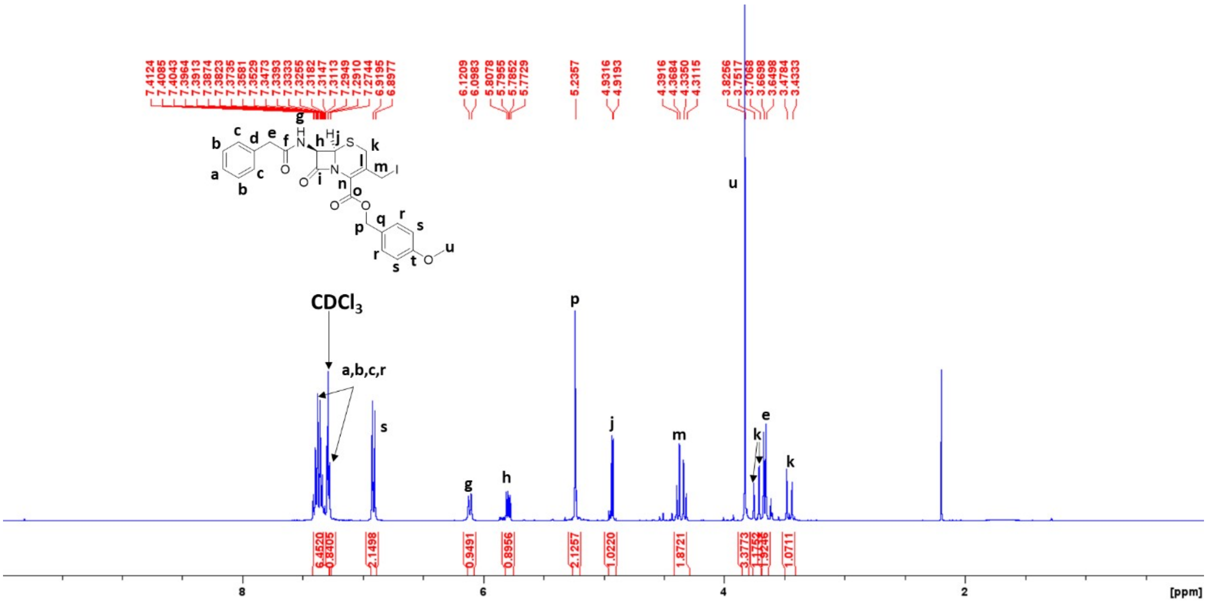

Figure S3. ^{1}H NMR spectra of compound **7** in $CDCl_3$.

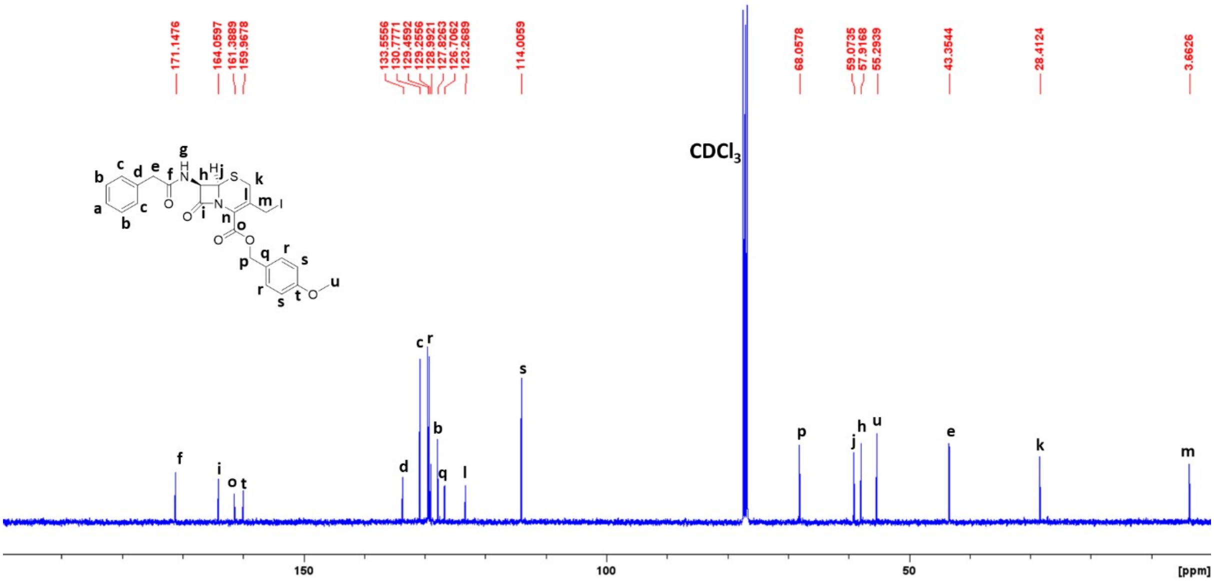

Figure S4. ^{13}C NMR spectra of compound **7** in $CDCl_3$.

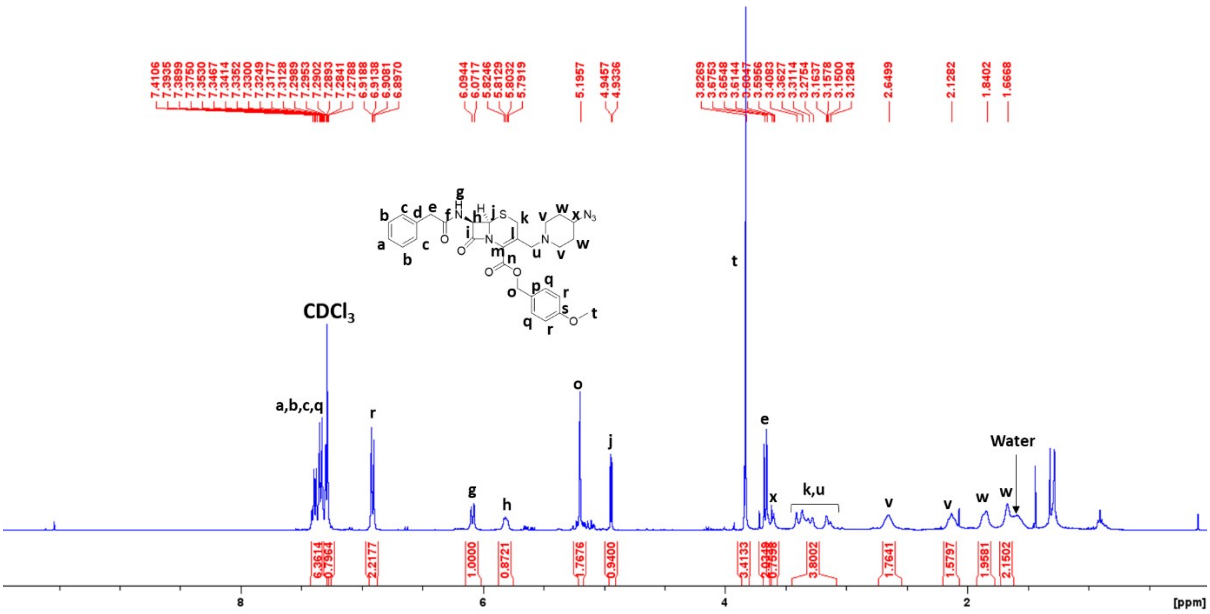

Figure S5. ^{1}H NMR spectra of compound **8** in $CDCl_3$.

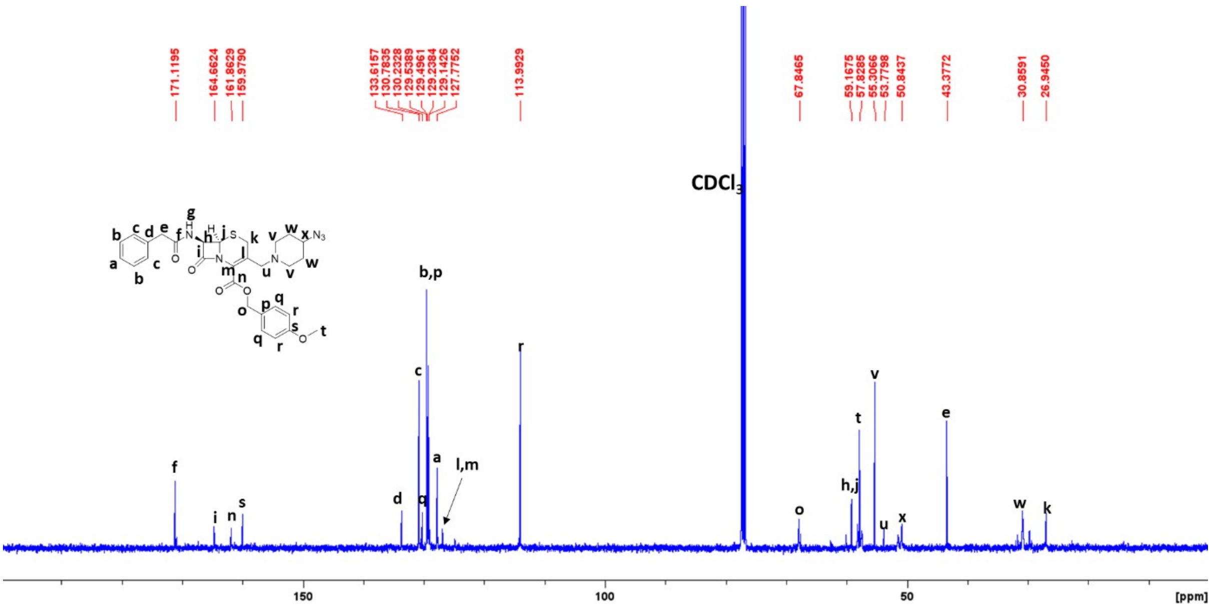

Figure S6. ^{13}C NMR spectra of compound **8** in $CDCl_3$.

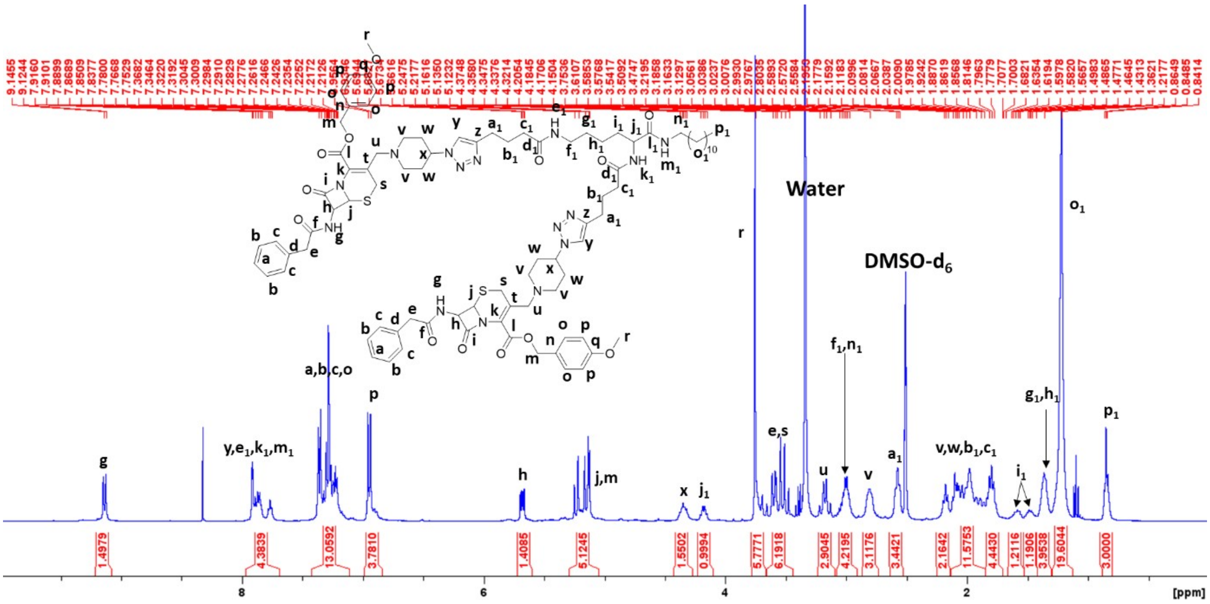

Figure S7. ^{1}H NMR spectra of compound **proBLM-Dendron** in DMSO-d_6.

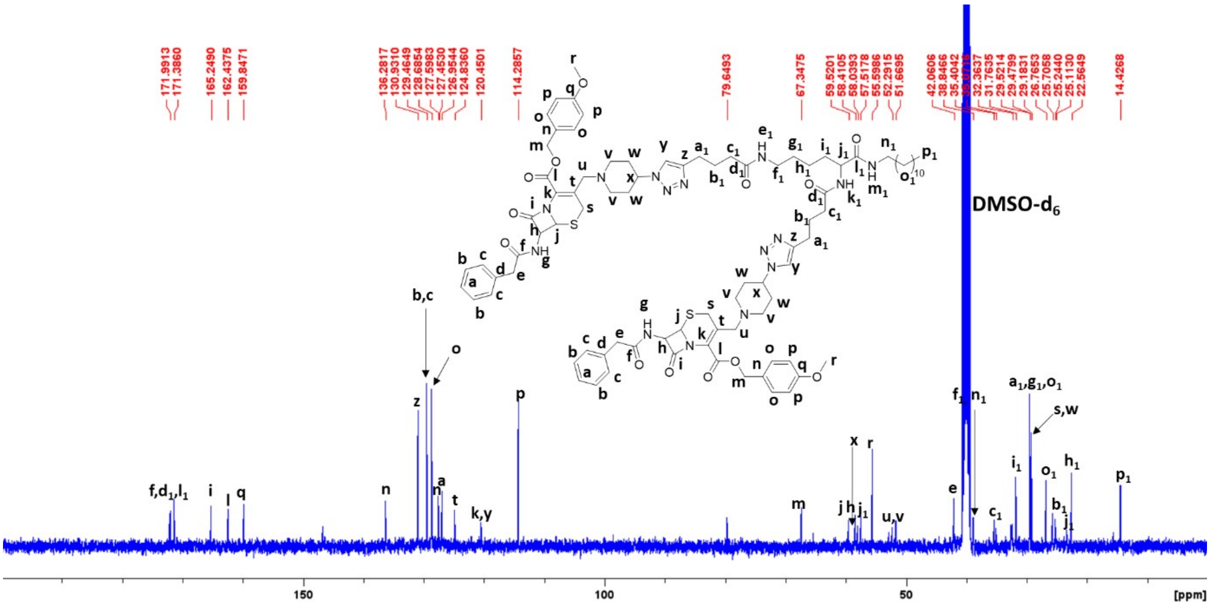

Figure S8. ^{13}C NMR spectra of compound **proBLM-Dendron** in DMSO-d_6.

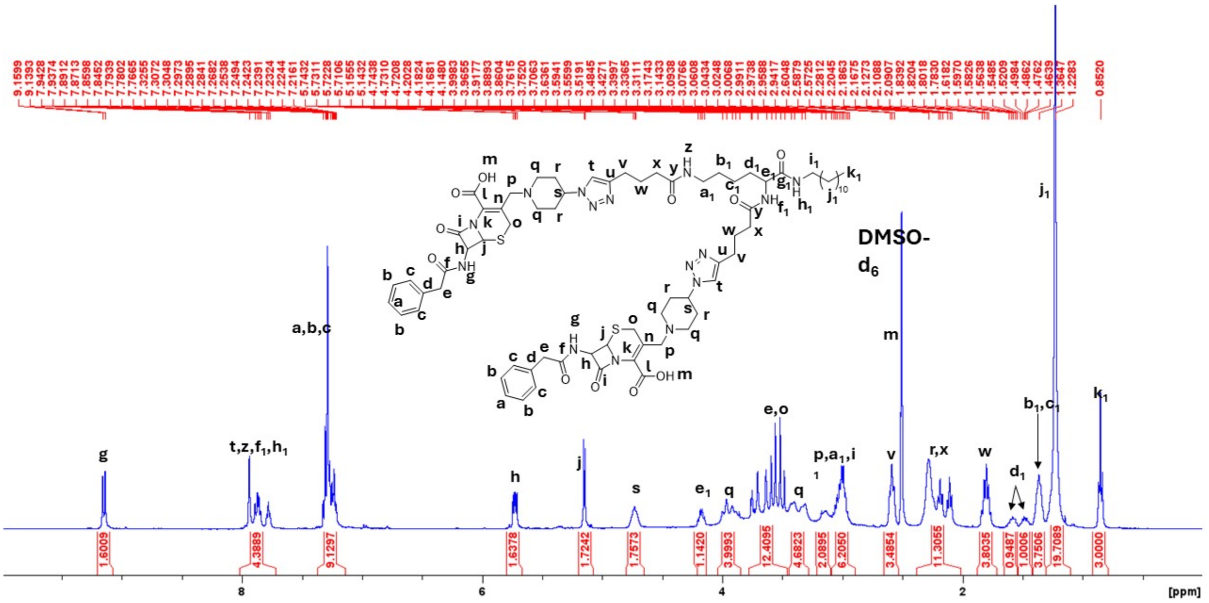

Figure S9. ^{1}H NMR spectra of **BLM-Dendron** in DMSO-d_6.

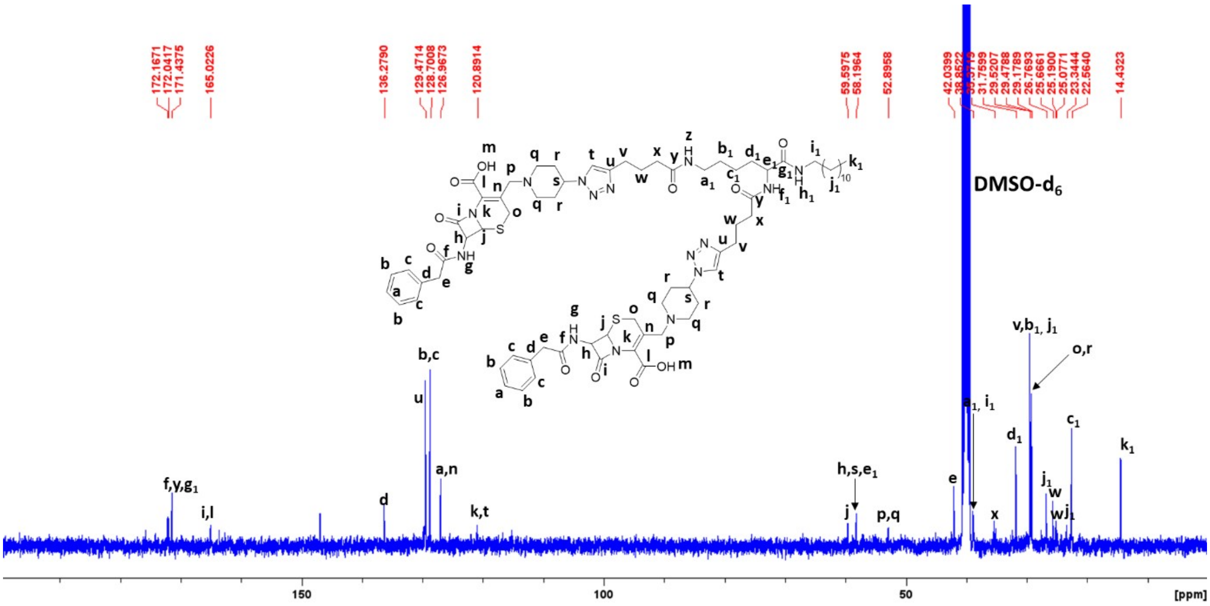

Figure S10. ^{13}C NMR spectra of **BLM-Dendron** in DMSO-d_6.

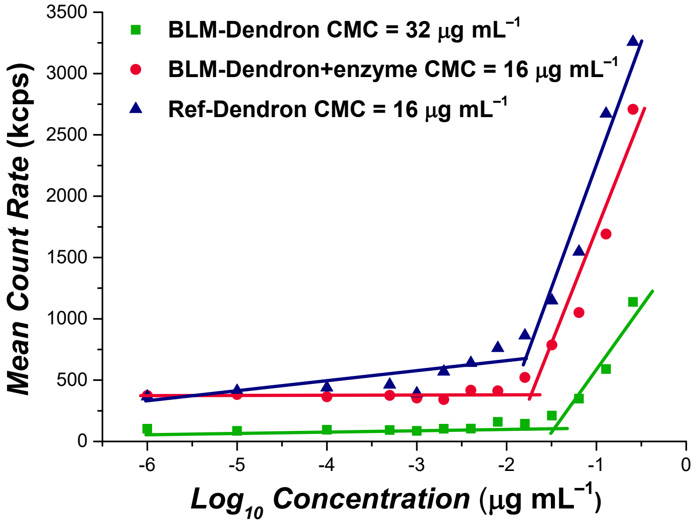

Figure S11. CMC estimation of compounds by plotting surface tension versus mean count rate.

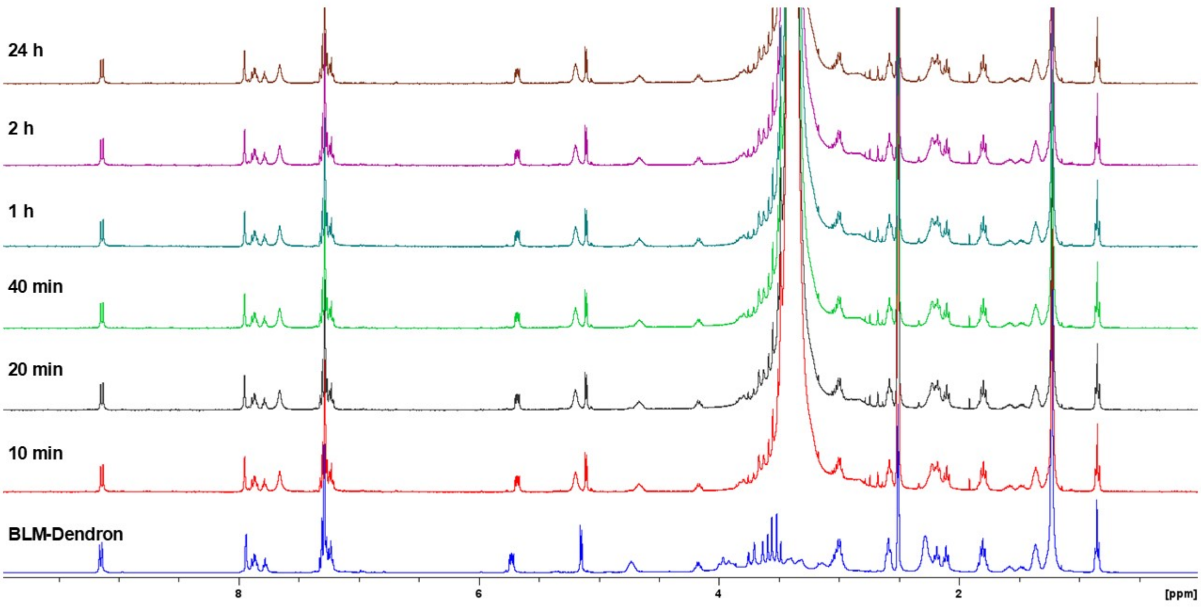

Figure S12. Uncaging kinetics of **BLM-Dendron** in the presence of penicillinase as monitored by ^{1}H NMR analysis in DMSO-d_6.

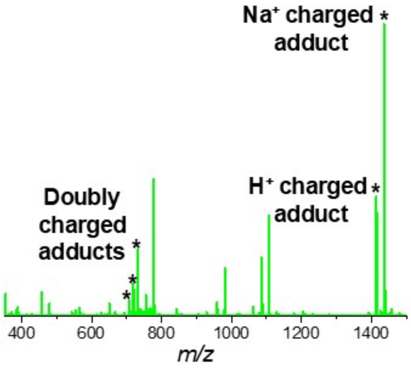

Figure S13. ESI-MS spectra of **BLM-Dendron** showing singly and doubly charged adducts as the main peaks. Note that the dendron undergo some fragmentation during ionization to yield half and fully uncaged dendrons.

REFERENCES

1. J.-M. Noy, F. Chen, D. T. Akhter, Z. H. Houston, N. L. Fletcher, K. J. Thurecht and M. H. Stenzel, Direct Comparison of Poly(ethylene glycol) and Phosphorylcholine Drug-Loaded Nanoparticles In Vitro and In Vivo, *Biomacromolecules*, 2020, **21**, 2320-2333.
2. M. Callari, P. L. De Souza, A. Rawal and M. H. Stenzel, The Effect of Drug Loading on Micelle Properties: Solid-State NMR as a Tool to Gain Structural Insight, *Angew. Chem. Int. Ed.*, 2017, **129**, 8561-8565.
3. Z. Y. Shao, Y. D. Xu, H. Luo, K. Hakobyan, M. N. Zhang, J. T. Xu, M. H. Stenzel and E. H. H. Wong, Smart Galactosidase-Responsive Antimicrobial Dendron: Towards More Biocompatible Membrane-Disruptive Agents, *Macromol. Rapid Comm.*, 2024, **45**, 2400350.
4. Z. Y. Shao, H. Luo, T. H. Q. Nguyen and E. H. H. Wong, Effects of Secondary Amine and Molecular Weight on the Biological Activities of Cationic Amphipathic Antimicrobial Macromolecules, *Biomacromolecules*, 2024, **25**, 6899-6912.
5. G. Y. Zhao, M. J. Miller, S. Franzblau, B. J. Wan and U. Möllmann, Syntheses and studies of quinolone-cephalosporins as potential anti-tuberculosis agents, *Bioorg. Med. Chem. Lett*, 2006, **16**, 5534-5537.
6. Z. Y. Shao, K. Hakobyan, J. T. Xu, R. X. Chen, N. Kumar, M. Willcox and E. H. H. Wong, Photoinduced Unveiling of Cationic Amine: Toward Smart Photoresponsive Antimicrobial Polymers, *ACS Appl. Polym. Mater.*, 2023, **5**, 8735-8743.
